# Epigenetic landscape in the kick-and-kill therapeutic vaccine BCN02 clinical trial is associated with antiretroviral treatment interruption (ATI) outcome

**DOI:** 10.1016/j.ebiom.2022.103956

**Published:** 2022-03-21

**Authors:** Bruna Oriol-Tordera, Anna Esteve-Codina, María Berdasco, Míriam Rosás-Umbert, Elena Gonçalves, Clara Duran-Castells, Francesc Català-Moll, Anuska Llano, Samandhy Cedeño, Maria C. Puertas, Martin Tolstrup, Ole S. Søgaard, Bonaventura Clotet, Javier Martínez-Picado, Tomáš Hanke, Behazine Combadiere, Roger Paredes, Dennis Hartigan-O'Connor, Manel Esteller, Michael Meulbroek, María Luz Calle, Alex Sanchez-Pla, José Moltó, Beatriz Mothe, Christian Brander, Marta Ruiz-Riol

**Affiliations:** a IrsiCaixa, AIDS Research Institute, Institute for Health Science Research Germans Trias i Pujol (IGTP), Hospital Germans Trias i Pujol, 2nd floor, Ctra del canyet s/n, Badalona, Barcelona 08916, Spain; b Departament de Biologia Cel·lular, de Fisiologia i d'Immunologia, Universitat Autònoma de Barcelona, Av. de Can Domènech 737, Cerdanyola del Vallès, Barcelona 08193, Spain; c Centro Nacional de Análisis Genómico (CNAG), Barcelona Science Park - Tower I, Carrer de Baldiri Reixac 4, Barcelona 08028, Spain; d Universitat Pompeu Fabra (UPF), Plaça de la Mercè 10-12, Barcelona 08002, Spain; e Cancer Epigenetics and Biology Program (PEBC), Bellvitge Biomedical Research Institute, Vinguda de la Granvia de l'Hospitalet 199, L'Hospitalet de Llobregat, Barcelona 08907, Spain; f Epigenetic Therapies Group, Experimental and Clinical Hematology Program (PHEC), Josep Carreras Leukaemia Research Institute, Ctra de Can Ruti - Camí de les Escoles, s/n, Badalona, Barcelona 08916, Spain; g Department of Clinical Medicine - Department of Infectious Disease, Aarhus University Hospital, Palle Juul-Jensens Boulevard 99, East Jutland, Aarhus 8200, Denmark; h Centre d'Immunologie et des Maladies Infectieuses – Paris (Cimi-Paris), INSERM U1135, Sorbonne Université, Bd de l'Hôpital 91, Paris, Île de France 75013, France; i CIBERINFEC, Madrid, Spain; j Fundació Lluita contra la Sida, Infectious Diseases Department, Hospital Universitari Germans Trias i Pujol, Ctra del Canyet s/n, Badalona, Barcelona 08916, Spain; k Centre for Health and Social Care Research (CESS), Faculty of Medicine, University of Vic - Central University of Catalonia (UVic - UCC), Carrer Miquel Martí i Pol, 1, Vic, Barcelona 08500, Spain; l Institució Catalana de Recerca i Estudis Avançats (ICREA), Passeig de Lluís Companys, 23, Barcelona 08010, Spain; m The Jenner Institute, University of Oxford, Old Road Campus Research Build, Roosevelt Dr, Headington, Oxford, Oxfordshire OX3 7DQ, UK; n Joint Research Center for Human Retrovirus Infection, Kumamoto University, Honjo 2-2-1, Kumamoto City, Chuo-ku 860-0811, Japan; o Department of Medical Microbiology and Immunology, University of California, Veterinary Medicine 3A, Davis, CA 95616, USA; p Division of Experimental Medicine, UC Davis School of Medicine, 4610 X Street, Sacramento, CA 95817, USA; q Cancer and Leukemia Epigenetics and Biology Program (PEBCL), Josep Carreras Leukaemia Research Institute, Ctra de Can Ruti - Camí de les Escoles, s/n, Badalona, Barcelona 08916, Spain; r Centro de Investigacion Biomedica en Red Cancer (CIBERONC), Av. Monforte de Lemos 3-5. Pabellón 11. Planta 0, Madrid 28029, Spain; s Department of Physiological Sciences II, School of Medicine, University of Barcelona, Feixa Llarga, s/n, L'Hospitalet de Llobregat, Barcelona 08907, Spain; t Projecte dels NOMS-Hispanosida, BCN Checkpoint, Carrer del Comte Borrell, 164-166, Barcelona 08015, Spain; u Biosciences Department, Faculty of Sciences and Technology, University of Vic-Central University of Catalonia, Carrer de la Laura 13 – Torre dels Frares, Vic, Barcelona 08500, Spain; v Statistics Department, Biology Faculty, University of Barcelona, Diagonal 643, Barcelona 08028, Spain; w Statistics and Bioinformatics Unit Vall d'Hebron Institut de Recerca (VHIR), Passeig de la Vall d'Hebron, 129, Barcelona 08035, Spain

**Keywords:** HIV-1 vaccine, Epigenetics, DNA methylation

## Abstract

**Background:**

The BCN02-trial combined therapeutic vaccination with a viral latency reversing agent (romidepsin, RMD) in HIV-1-infected individuals and included a monitored antiretroviral pause (MAP) as an efficacy read-out identifying individuals with an early or late (< or > 4weeks) viral-rebound. Integrated -omics analyses were applied prior treatment interruption to identify markers of virus control during MAP.

**Methods:**

PBMC, whole-genome DNA methylation and transcriptomics were assessed in 14 BCN02 participants, including 8 Early and 4 Late viral-rebound individuals. Chromatin state, histone marks and integration analysis (histone-3 acetylation (H3Ac), viral load, proviral levels and HIV-specific T cells responses) were included. REDUC-trial samples (*n* = 5) were included as a control group for RMD administration alone.

**Findings:**

DNA methylation imprints after receiving the complete intervention discriminated Early versus Late viral-rebound individuals before MAP. Also, differential chromatin accessibility and histone marks at DNA methylation level were detected. Importantly, the differential DNA methylation positions (DMPs) between Early and Late rebounders before MAP were strongly associated with viral load, proviral levels as well as the HIV-specific T-cell responses. Most of these DMPs were already present prior to the intervention and accentuated after RMD infusion.

**Interpretation:**

This study identifies host DNA methylation profiles and epigenetic cascades that are predictive of subsequent virus control in a kick-and-kill HIV cure strategy.

**Funding:**

European Union Horizon 2020 Framework Programme for Research and Innovation under Grant Agreement N°681137-EAVI2020 and N°847943-MISTRAL, the Ministerio de Ciencia e Innovación (SAF2017_89726_R), and the National Institutes of Health–National Institute of Allergy and Infectious Diseases Program Grant P01-AI131568.

**Research in context**

**Evidence before this study**

The lack of an effective HIV-1 cure is mainly due to the virus´ capacity to establish HIV-1 reservoirs within latently infected cells. To overcome this issue, the kick-and-kill strategy has been proposed as a way to disrupt the HIV-1 latency and to improve the antiviral immune response. The BCN02 study, combined a therapeutic HIV-1 vaccine with romidepsin, a latency reversing agent, and showed that some individuals maintain a low viral load for extended periods of time after antiretroviral treatment interruption (ATI). The understanding of the mechanisms and host factors driving such HIV-1 control could benefit new HIV-1 cure strategies. Since previous studies of gene expression profiles and DNA methylation have yielded promising imprints associated with natural HIV-1 control and potential therapeutic targets, a combined transcriptomics and epigenomics study was conducted in the context of the BCN02 study.

**Added value of this study**

In the present study, we determined that the administration of an HIV-1 therapeutic vaccine together with the latency reversing agent romidepsin has a profound impact on the host epigenomics and transcriptomics patterns, which ultimately affect genes involved in pathways related to HIV-1 infection (“*HIV life cycle*”), immune response (“*T-cell response*”, “*B cell response*” and “*innate immunity*”) and other relevant biological processes like cell proliferation, gene expression regulation and metabolism. Additionally, we identify that the epigenomic landscape after the whole intervention and before treatment interruption is associated with the differential viral rebound kinetics and is additionally correlated with HIV-1 proviral levels, peripheral blood viral loads and the strength of the HIV-1-specific T-cell responses, suggesting an important role of host epigenomic regulation to achieve an HIV-1 cure.

**Implications of all the available evidence**

Our findings, together with a recent article in individuals treated with a latency reversing agent (NCT01680094), which identified DNA methylation imprints associated with HIV-1 reservoir size and time to viral rebound after ATI, suggest that the inclusion of DNA methylation studies in future clinical studies might be relevant to identify predictors of viral control prior to the interruption of antiretroviral treatment. Additionally, a few studies have related HIV-1 infection, ART treatment, and HIV-1 control with DNA methylation marks, therefore a comprehensive study of the host epigenome in clinical intervention might be relevant to fine-tune such strategies to induce effective HIV cure.

Alt-text: Unlabelled box

## Introduction

Human immunodeficiency virus-1 (HIV-1) infection forms a viral reservoir during early stages of infection which makes lifelong antiretroviral treatment (ART) indispensable and presents the main barrier to an HIV-1 cure. Additionally, despite the effectiveness of ART, it is not available worldwide, it is costly and is associated with viral resistance and treatment-related side effects. The establishment of an HIV-1 reservoir along with sub-optimal and dysfunctional antiviral immune responses lays the basis of uncontrolled viral replication and HIV disease progression in the absence of ART. To this end, different kick-and-kill strategies are being designed to disrupt viral latency and enhance the host´s immune system to eliminate HIV-1-infected cells.^1^, ^2^, ^3^

A wide variety of latency reversing agents (LRAs) have been proposed to reactivate the viral reservoir, including epigenetic modifiers and agonists of NF-kB, Toll-Like-Receptor (TLR) and protein kinase C, among others.^4^ Histone deacetylase inhibitors (HDACi) like vorinostat, panabinostat or romidepsin (RMD), originally developed for other medical indications (cancer), were first to be translated to the HIV cure field.^4^^,^^5^ HDACi administration is associated with a global increase of histone acetylation that leads to chromatin relaxation and an increase in gene expression, including HIV transcripts, thus rendering virally infected cells visible to the immune system. However, LRA administration has generally failed to eliminate HIV-infected cells efficiently and no clinically relevant reductions in provirus loads have been observed.^4^^,^^6^^,^^7^ Given that HDACi were developed as anticancer drugs, they may hinder the effective clearance of reactivated virus-infected cells by a transient immune-suppression of CD8 T cell activity, whose effects would be cumulative with a generally dysfunctional CTL immune response due to viral infection.^8^, ^9^, ^10^ To overcome these limitations, clinical trials combining LRAs with therapeutic T cell vaccination aim to control rebounding HIV by intensifying the anti-HIV specific CTL responses.^11^, ^12^, ^13^ However, the effects of LRAs administration on non-viral, host genes involved in antiviral immunity are poorly understood, although they may play an important role in shaping effective vaccine-induced immunity.

BCN02 (NCT02616874) was a pilot study that combined the MVA.HIVconsv^14^ vaccination before and after the administration of three cycles of RMD in early treated individuals rolled-over from the BCN01 (NCT01712425) clinical trial.^13^^,^^15^ In BCN02, RMD led to a transient increase of HIV transcription and histone 3 acetylation levels (H3Ac) that were short-lived and returned to basal levels one week after the last RMD cycle.^13^ The BCN02 study also included a 32-week monitored antiretroviral pause (MAP) to evaluate virus control post-intervention. Of the 13 individuals eligible for MAP, 4 maintained viral loads < 2000 HIV RNA copies/mL for more than 4 weeks and of these, 3 completed the entire 32 weeks of MAP without the need to restart ART.^13^ To gain insight into the molecular processes modulated during the clinical study, especially those that can affect HIV-1-specific T-cell responses and virus reactivation, as well as to identify predictors of ATI outcome before ART interruption, a systems biology analysis was conducted combining whole-genome gene expression and DNA methylation assessments in PBMCs samples from 14 individuals enrolled in the BCN02 clinical trial at three different time points (Figure S1a).

Recent findings of specific DNA methylation patterns associated with the natural ability to control HIV replication *in vivo*,^16^^,^^17^ raised the questions as to whether stable methylation profiles prior to ART interruption could determine the treatment outcome, and whether kick-and-kill strategies would have the power to restore potentially epigenetically dysregulated host gene pathways important for virus control. Therefore, DNA methylation was evaluated together with gene expression data in the context of the BCN02 trial. These results revealed the main pathways that were modulated by the cumulative effect of the vaccination and RMD, as well as their impact on chromatin states and histone marks. Despite the small number of participants, specific DNA methylation imprints in pre-MAP samples that discriminate Early (*n* = 8) or Late (*n* = 4) rebounding individuals were identified. Such marks were related to relevant pathways for virus infection (*HIV infection, HIV life cycle and Host interactions of HIV infection)*, and to immune signatures (*TCR signalling, MHC-II antigen presentation* and *signalling by interleukins)*; and mapped to regions with differential chromatin relaxation states and transcription factor binding sites (TFBS). Future analyses in larger clinical trials, ideally double-blinded, and placebo-controlled, will be needed to confirm these results and to test the use of DNA methylation signatures as a predictive tool for viral rebound before treatment interruptions are initiated.

## Methods

### Study design

BCN02 (NCT02616874) was a pilot kick-and-kill study that included 15 early-treated individuals who were rolled-over from the prior clinical trial BCN01 (NCT01712425) (Table S1).^15^ In BCN01, individuals were immunized with a heterologous prime/boost regimen delivering the HIVconsv immunogen. In BCN02, the participants were vaccinated again with MVA.HIVconsv before and after the administration of RMD (1 week after 3 cycles of RMD infusion) (Figure S1a). The BCN02 included a monitored antiretroviral pause (MAP) to evaluate the viral rebound kinetics whereby participants were monitored for viral load rebounds on a weekly basis for up to 32 weeks. Two participants were excluded from MAP evaluations, one who did not meet the criteria for treatment interruption, and one who showed a protocol deviation during this phase. Specifically, the present omics sub-study was based on the 14 male individuals enrolled in the BCN02, of which 12 were eligible for MAP. These 12 individuals were classed in Early rebound (*n* = 8) or Late rebound group (*n* = 4) according if they reached the threshold of 2000 HIV RNA copies/mL and restart of ART before or after the 4 weeks of treatment interruption.

In order to evaluate the contribution of RMD-only treatment on gene expression profiles in isolated CD4 T cells and whole PBMCs, samples from the part A of the clinical trial REDUC (NCT02092116)^6^ were used. REDUC was designed to evaluate the *in vivo* effect of RMD administration alone (1 week after 3 cycles of RMD infusions) in ART-treated individuals and showed that RMD could disrupt latency and led to a 2.4 to 5 fold increased HIV-1 transcription (cell-associated un-spliced HIV-1 RNA). Here, we included 5 participants from this clinical trial, sampled at 6 weeks before (Baseline) and 1 week after RMD administration, consistent to the same timepoints used in BCN02 study (Table S2). The sample size was determined based on sample availability of the BCN02 and the REDUC studies, respectively.

### PBMCs and CD4 T cell isolation and acid nucleic extraction

Blood samples from BCN02 participants were processed using Lymphoprep (STEMCELL technologies) and 2 × 10^6^ dry pelleted PBMCs were frozen until use. DNA and RNA were isolated simultaneously from the same sample (AllPrep DNA/RNA Mini Kit, Qiagen) and were frozen until use. PBMC's were isolated from blood samples from REDUC trial participants using Ficoll separation followed by CD4 T-cells isolation using a CD4 T-cell isolation kit and magnetic-activated cell sorting (MACS) columns (Miltenyi Biotec, purity >95%). Isolated CD4 cells were lysed using AllPrep lysis buffer and lysates were stored at -80°C until RNA was extracted (AllPrep isolation kit, Qiagen).

### RNAseq library preparation and sequencing and data pre-processing

The total RNA was quantified by Qubit® RNA BR Assay kit (Thermo Fisher Scientific) and the RNA integrity was estimated by using RNA 6000 Nano Bioanalyzer 2100 Assay (Agilent). The RNASeq libraries from total PBMC RNA samples were prepared using a TruSeq™ Stranded Total RNA kit protocol (Illumina) and the rRNA was depleted from 20 to 500 ng of total RNA using the RiboZero Magnetic Gold Kit. The libraries were sequenced on HiSeq2000 (Illumina) in paired-end mode with a read length of 2 × 76 bp using TruSeq SBS Kit v4 in a fraction of a sequencing v4 flow cell lane, following the manufacturer's protocol. Image analysis, base calling and quality scoring of the run were processed using the manufacturer's software Real Time Analysis (RTA 1.18.66.3) and followed by generation of FASTQ sequence files by CASAVA. RNA-seq paired-end reads were mapped against the human (GRCh38) genome using STAR version 2.5.3a^18^ with ENCODE parameters. Annotated genes (genecode version 28) were quantified using RSEM version 1.3.0 with default parameters.^19^ The quality control of the mapping and quantification steps was performed with GEMtools (https://gemtools.github.io/) and custom python scripts. Following RSEM expected counts were normalized with TMM method from edgeR R/Bioconductor and genes with less than 5 counts were filtered out.^20^ Finally, counts were transformed with the voom function in limma R/Bioconductor and used in downstream analyses.^21^

### DNA methylation array

Genomic DNA was bisulfite converted using EZ DNA methylation Kit (Zymo) following the manufacturer's protocol. Next, 4uL of bisulfite-converted DNA were hybridized to Infinium HumanMethylation450 BeadChip following Illumina Infinium HD Methylation protocol. For samples obtained during MAP, Infinium MethylationEPIC BeadChip kit was used. Chip analysis was performed using Illumina HiScan SQ fluorescent scanner and the intensities of the images were extracted using GenomeStudio (2010.3) Methylation module (1.8.5) software. Quality control, background correction and quantile normalization across arrays was performed using ChAMP R/Bioconductor package with functions extracted from Minfi R/Bioconductor package.^22^, ^23^, ^24^

### Differential gene expression and DNA methylation analysis

In exploratory data analysis of both -omics data sets, MDS (Multidimensional scaling) plots were used to identify batch effects and the major source of variation. In the DNA methylation dataset, the only female participant appeared as an outlier in the MDS plot. Additionally, the impossibility to take sex variation into account due to the presence of a single female in the study, this participant was removed from the omics substudy (Figure S1b). BCN02 was conducted across two hospitals in the Barcelona metropolitan area and biological samples were processed at the respective laboratories. The sample processing laboratory parameter was also identified as a source of variation in gene expression data (Figure S1c). Therefore, ComBat function from SVA R/Biconductor was used to correct for this effect in unsupervised analysis.^25^ Differential gene expression and methylation analyses between time points and Early and Late rebounders were performed with limma.^21^ For longitudinal comparisons, ‘duplicateCorr’ function was used to consider the participants variable as a random effect. To identify the changes in gene expression over time, the model ∼*Laboratory*+*Week* was fitted, to adjust for the confounding variable *Laboratory*. For DNA methylation (M-values), the model ∼*Week* was used. Next, to identify the individuals with an Early (*n* = 8) or Late (*n* = 4) rebound in the different time points, the fitted model was ∼*Week_Rebound.*

### Gene expression and DNA methylation profiles

Soft clustering (MFuzz R/Bioconductor) allowed discerning different longitudinal clusters of gene expression or DNA methylation. Briefly, genes or CpG positions that showed a *p*-value < 0.05 in any of the pairwise comparison between time points were included. Next, for each gene in each of the time points, the median expression/methylation standardized value was used for clustering. The function MFuzz:mestimate() was used to estimate the fuzzier parameters, and the number of clusters was determined with the MFuzz::Dmin() function. Finally, a minimal membership of 0.1 was used for gene expression, and of 0.2, for DNA methylation.

### Integrated functional pathways analysis of genes and CpG positions modulated during the kick-and-kill therapy

For integration of the differentially expressed genes (DEGs, *p*-value < 0.05) and differentially methylated positions (DMP *p*-value < 0.05) data sets corresponding to one week after RMD administration (Vacc+RMD) and the baseline (BSL) (Figure S1), a published protocol was followed.^26^ For functional analyses, a pre-ranked GSEA was run in GSEA JAVA desktop program (MacOSX Version 4.03). Genes were ranked according to the –log10 *p*-value multiplied by the sign of log2 Fold-Change.^26^ As database, a combination of the REACTOME subset of Canonical pathways from Molecular Signature database (MSigDB, v7.4), and the blood transcriptional modules (BTMs) previously described elsewhere^27^ were used. The majority of parameters was set to default (1000 permutations, weighted enrichment statistic and only gene sets between 15 and 500 genes were considered). Combined GSEA results for gene expression and DNA methylation were plotted using EnrichmentMap^27^ in Cytoscape (MacOSX version 3.7.3). The different nodes represent the pathways, and the edges show the mutual overlap. The largest pathway was maintained when there were one or more pathways with similar names, one embedded inside the other. BTMs annotations in the miscellaneous groups TBC were as well removed.^28^ Finally, groups of pathways were done using AutoAnnotate^29^ and modified according to authors criteria based on REACTOME data base and PubMed bibliographic research and considering the context of the study and the sample type (PBMCs).

### Chromatin state and histone marks enrichment

To identify the chromatin regions and histone marks in which DEGs or DMPs were enriched, either based on differences between time points or between early or late rebound, a genomic regions enrichment was run with LOLA R/Bioconductor^30^ based on the publicly available Roadmap datasets of 18-chromatin states (ChromHMM) and histone marks of PBMCs (E062, https://egg2.wustl.edu/roadmap/web_portal/chr_state_learning.html). As background, non-differentially expressed genes or the non-differentially methylated CpGs positions were used.

### Transcription factor (TF) enrichment

To determine the enrichment of the TF binding site motifs in DMPs between Early and Late rebound (at the Vacc+RMD time point), HOMER motif discovery software was used,^31^ considering a 250 bp window upstream and downstream of the DMPs. The other CpGs in the 450K array were used as background.

### Evaluation of DNA methylation and gene expression capacity to discriminate between early and late rebound

To test the capacity of DNA methylation and gene expression to differentiate Early or Late rebound at the different time points analysed, a Principal Component Analysis (PCA) in each of the datasets for each time point was used, and a logistic regression was fitted with PC1 and PC2 as predictors. Area Under de Curve (AUC) values showed how well the PC1 and PC2 classified the Early or Late rebounder groups. Based on the DMPs between Early and Late Rebound at Vacc+RMD time point, a GSEA analysis was applied using canonical pathways and BTMs as described in methods above. Based on GSEA results, 6 modules of DMPs were identified, and the Z-score methylation levels (M-values) of DMPs on genes in these pathways were represented in a heatmap (ComplexHeatmap R/Bioconductor).^32^ Additionally, the Rho values (Spearman Correlation) of DMPs with specific viral (HIV viral load and proviral levels) parameters, HIV-1-specific T-cell responses (measured by INFg ELISpot) and Histone H3 acetylation levels were plotted.

### Determination of viral parameters

Peripheral blood proviral levels (CA-HIV-1 DNA) were measured by ddPCR in isolated CD4 T cells. Proviral DNA and cell-associated HIV-1 RNA (CA-HIV-1 RNA) were both assessed using two different sets of primers/probe annealing to 5’LTR and Gag. The RPP30 housekeeping gene was used for genomic DNA normalization while TBP housekeeping gene before RMD administration was used for gene expression normalization. To determine the HIV-1 RNA plasma levels below 20 copies/ml ultrasensitive viral load was performed with 4–8 ml plasma samples from participants, ultra-centrifuged (170,000xg, 4 °C, 30 min) prior to RNA viral extraction (m2000sp Abbot device). Abbott Real-Time HIV-1 assay was used to determine HIV-1 RNA copies.

### IFNg ELISpot assay

T-cell responses against HIV-1 or HIVconsv were assessed by IFNg ELISpot as described in the previous BCN02 main study report.^13^ Briefly, thawed cryopreserved PBMC from samples drawn at different time points were cultured in duplicate in 96-well ELISpot plates (MultiScreen HTS MSIPS4W10, Millipore) and stimulated with pools of 15-mer peptides overlapping peptides covering the HIVconsv immunogen (HIVconsv, 6 pools of 32-33 peptides per pool) or the regions of HIV-1 not covered by the immunogen (12 pools of 39–47 peptides) as described.^13^ Medium without peptides was used as negative control, and as positive control the cells were stimulated with PHA and the commercially available CEF peptide pool (C.T.L OH, USA). The threshold to consider a positive T-cell response was the maximum value of 3 determinations: 50 SFC (Spots Forming Cells)/10^6^ PBMC (5 spots per well), > the mean of SFC in negative control wells plus 3 standard deviation of the negative control wells, or > 3 × the mean of negative control wells. The HIVtotal T-cell magnitude was determined as the sum of SFC/10^6^ PBMC for all positive responses, the HIVtotal breadth was calculated as the sum of all the positive responses. The same was applied to the responses targeting the regions covered by the HIVconsv immunogen to determine the magnitude and breadth of the HIVconsv specific T-cell response.

### Histone H3 acetylation (H3Ac)

Cryopreserved PBMCs were used to evaluate the H3Ac in lymphocytes by flow cytometry as described previously^13^. Briefly, after a 20 min blocking with PBS 10% FBS, cells were stained for 30 min with anti-acetyl histone H3 polyclonal rabbit (MerckMillipore 06–599) or with the control stain using normal rabbit serum (LifeTechnologies 10510). After washing, a 30 min incubation with the secondary antibody in the dark was performed (donkey anti-rabbit IgG, LifeTechnologies A21206). The median fluorescence intensity (MFI) for acetyl histone H3 stain was determined and the background was subtracted using the MFI of the isotype control staining.

### Statistics

The moderated t-test in limma was used to identify the differentially methylated positions or the differentially expressed genes between different time points or between Early and Late rebound. Although false discovery rate (FDR) adjusted *p*-value was calculated for each gene transcript or CpG position, due to the small sample size and the exploratory character of the present study, an uncorrected *p*-value < 0.05 was used to define the subset of differentially expressed genes (DEGs) and differentially methylated positions (DMPs). Chi-square tests were applied to evaluate the different abundance of DMPs or DEGs in certain chromosomes in contrast to the evaluated CpG positions or transcripts. The same test was applied for DMPs in relation to island (island, open sea, shore or self) or in relation to gene (5’UTR, TSS200, TSS1500, 1stExon, Body, 3’UTR) and for DEGs in relation to biotypes. For chi-square test, a *p*-value < 0.05 was considered significant. Finally, Spearman's rank correlation test was used in correlation analysis and a *p*-value < 0.05 was considered significant.

### Ethics

The study of all samples used in the present study was approved by the Clinical Research Ethics Committee of the Germans Trias i Pujol University Hospital (Reference number PI-18-183) and all the participants signed an informed consent.

### Data deposition and material sharing

Transcriptomics and DNA Methylation is uploaded at Gene Expression Omnibus (GEO): GSE184653, contains the transcriptomics data for BCN02 study; GSE185391, the DNA Methylation data for BCN02 study; and GSE185027, the transcriptomics data for the individuals from the REDUC study. The R code used in the analysis can be found in the GitHub https://github.com/hostimmuneOMICS under the repository BCN02_OmicsAnalysis.

### Role of the funding source

This publication has received funding from the European Union Horizon 2020 Framework Programme for Research and Innovation under Grant Agreement N° 681137-EAVI2020 and N° 847943-MISTRAL, the Ministerio de Ciencia e Innovación (SAF2017_89726_R), and the National Institutes of Health – National Institute of Allergy and Infectious Diseases Program Grant P01-AI131568. The funding sources had no role in writing the manuscript nor in data collection, analysis and interpretation or any other aspect of the study. Funding sources only require to submit the publication in open access.

## Results

### Gene expression and DNA methylation changes one week after MVA.HIVconsv vaccination in the BCN02 study

**Figure 1:**
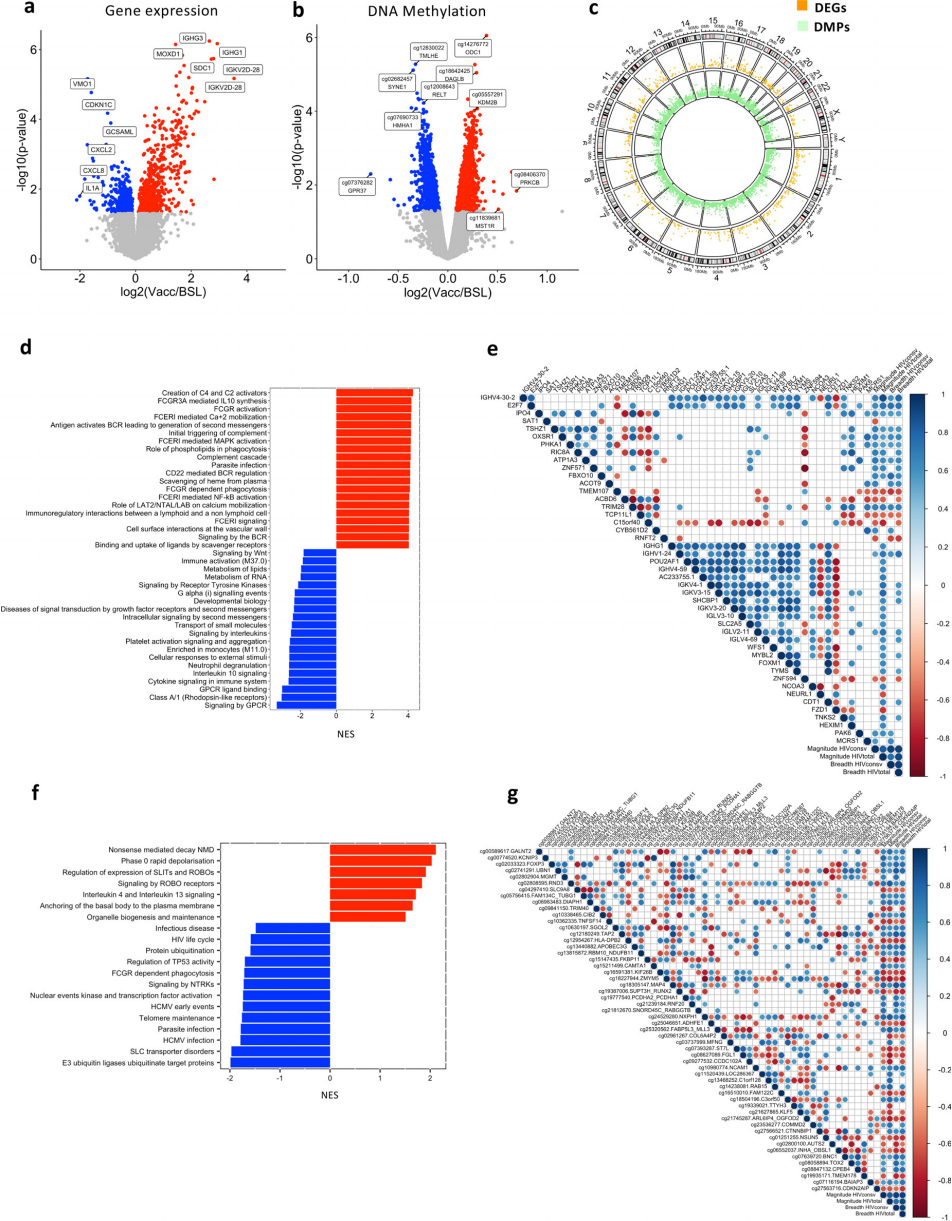
**Impact of vaccination on gene expression and DNA methylation in the BCN02 trial**. (a) and (b) show the Volcano Plots for the 1287 DEGs and the 5447 DMPs between post-vaccination and baseline, respectively (*p*-value < 0.05). *X*-axis show the log2 Fold-Change, and Y-axis the -log10 *p*-value. Grey colour indicates *p*-value > 0.05; red, *p*-value < 0.05 and FC > 0 and blue, *p*-value < 0.05 and FC < 0. (c) CIRCOS plot that includes an outer track with the chromosomes, genes and CpG positions in the genome. The two inner tracks are the Manhattan plots for DMPs (green) and DEGs (orange) between post-vaccination and baseline time points. (d) and (f) show the pathway enrichment (GSEA) for DEGs and DMPs, respectively. Red colour reflects NES > 0 and blue, NES < 0. (e) Correlation plots of DEGs with breadth and magnitude of the virus vaccine-specific T-cell response evaluated by IFNg ELISpot as detailed in the methods section (*p*-value < 0.05 & Rho > |0.6|). For both, DEGs and magnitude and breadth of the T-cell response, the log2 change between vaccination and baseline was used. (g) Correlation plot for DMPs and T-cell responses (*p*-value < 0.01 & Rho > |0.6|) against the HIVconsv immunogen or total HIV-1. For both, DMPs and magnitude and breadth of the T cell response, the log2 change between vaccination and baseline was used. Blue indicates positive and red indicates negative Rho values (Spearman's correlation). Blanks make reference to non-significant correlations.

To evaluate the impact of the first MVA.HIVconsv vaccination (Vacc) on the host transcriptional and epigenetic signatures, whole genome gene expression and DNA methylation screenings were conducted. Multidimensional scaling analysis (MDS) and volcano plot of data from samples drawn one week after the HIV.consv vaccination, revealed a strong impact on the host gene transcriptional program (Figures S2a and 1a). Specifically, compared to basal time point, 673 upregulated (Figure 1a, red) and 524 downregulated (Figure 1a, blue) genes were identified (*p*-value < 0.05, limma model, Table S3). After adjusting for multiple comparisons with false discovery rate (FDR), there were 95 DEGs (adjusted *p*-value < 0.1), of which 88 were upregulated and 7 downregulated. The majority of differentially expressed genes (DEGs, *p*-value < 0.05, limma model) were protein coding genes (67%) and 7%, immunoglobulin coding genes (Table S4). In parallel, 5447 differentially methylated positions (DMPs, *p*-value < 0.05, limma model, Table S5) were identified, that were symmetrically distributed in hypermethylated (*n* = 2864, Figure 1b red) and hypomethylated DMPs (*n* = 2583, Figure 1b blue) one week after MVA.HIVconsv vaccination, as shown in the volcano plot (Figure 1b and S2b). After FDR adjustment only 2 hypermethylated DMPs remained significant (adjusted *p*-value < 0.1). Of the identified DMPs (*p*-value < 0.05, limma model), 57% mapped to regulatory regions (TSS200, TSS1500, 1stExon and 5’UTR regions), 38% to Body regions, and 5% to 3’UTR regions (Figure S1c, and Table S6). Overall, 40% of DMPs were located in CpG islands, while 22% were from islands shores and 7%, from island shelves. The remaining 32% of DMPs, were found to open sea regions (Figure S1d, and Table S6).

While DEGs and DMPs between Vacc and baseline time points were widely distributed among the different chromosomes (Figure 1c), the highest proportion of DEGs and DMPs were found in chromosome 1 (chr1), which is the largest chromosome. Overall, DEGs and DMPs in each chromosome were proportional to its length (Figure S1e, f, Tables S4–S6). In DNA methylation, chr8 and chrX showed a significant reduction in the proportion of DMPs in comparison to CpG positions that are included in the array (Figure S1e and Table S6).

At pathway and blood transcription modules (BTMs) level, based on DEGs, the majority of genes that were upregulated after vaccination (Normalized Enrichment Score, NES > 0) were related to Fc receptors, complement activation, phagocytosis and B cell receptor (BCR) activation. In parallel, the majority of downregulated genes (NES < 0) were linked to pathways of GPCR (G protein-coupled receptors) as well as interleukin and cytokine signalling (Figure 1d). Correlation analysis of DEGs belonging to these categories were associated with the magnitude of the IFNg T-cell responses against total HIV-1 (including the sum of HIVregions in plus out of HIVconsv), while fewer were correlated with IFNg T-cell responses against HIVconsv (Figure 1e). Of these, MCRS is a gene involved in chromatin organization, and the genes SAT1, RNFT2 and ACOT9 are all involved in metabolic processes.^33^^,^^34^

DNA methylation pathway analyses showed an overall negative enrichment score, with the majority of the gene-annotated DMPs being hypomethylated after vaccination. These DMPs were mainly involved in infection-related pathways (e.g. *HIV life cycle, HCMV early events*) (Figure 1f). In parallel, pathways like *interleukin 4 and 3 signalling* and *SLITs and ROBOs signalling* contained mainly hypermethylated DMPs (NES > 0). Interestingly, the Slits2 protein has been described as an inhibitor of HIV-1 replication in T cells.^35^ When assessing correlations between DMPs and the total T-cell responses against entire HIV or HIVconsv measured by IFNg ELISpot,^13^ the methylation levels of 32 CpG positions showed a positive correlation with these parameters, while 22 were negatively correlated. Of relevance, the methylation levels of *APOBEC3G*, involved in HIV-1 antiviral function, and *TAP2*, a gene involved in antigen presentation were both positively correlated with HIV total and HIVconsv T-cell breadth and magnitude. Of the DMPs positively correlated with T-cell response magnitude against HIVconsv but not total HIV, the majority of associated genes were involved in chromatin regulation and gene transcription (e.g. *MGMT, RNF20, RABGGTB, TOX2* and *CPEB.*^33^^,^^34^ Finally, the methylation levels of *AUTS2* gene, associated with the chromatin remodelling complex PRC1, were negatively correlated with the magnitude of the HIVconsv specific T-cell response.^33^^,^^34^

These data demonstrate a strong impact of vaccination on the gene transcriptional program, particularly for immunoglobulins genes and for immune response pathways including Fc receptors, interleukins and cytokines and complement signalling. The same intervention impacted DNA methylation levels of genes involved in responses to infection. Interestingly, DNA methylation imprints correlated with measures of the HIVconsv vaccine-specific T cell immunity, suggesting a direct role of epigenetic modulation on vaccine responsiveness or an impact of vaccination on epigenetic cascades.

### Cumulative transcriptional and methylation changes after vaccination and RMD intervention

**Figure 2:**
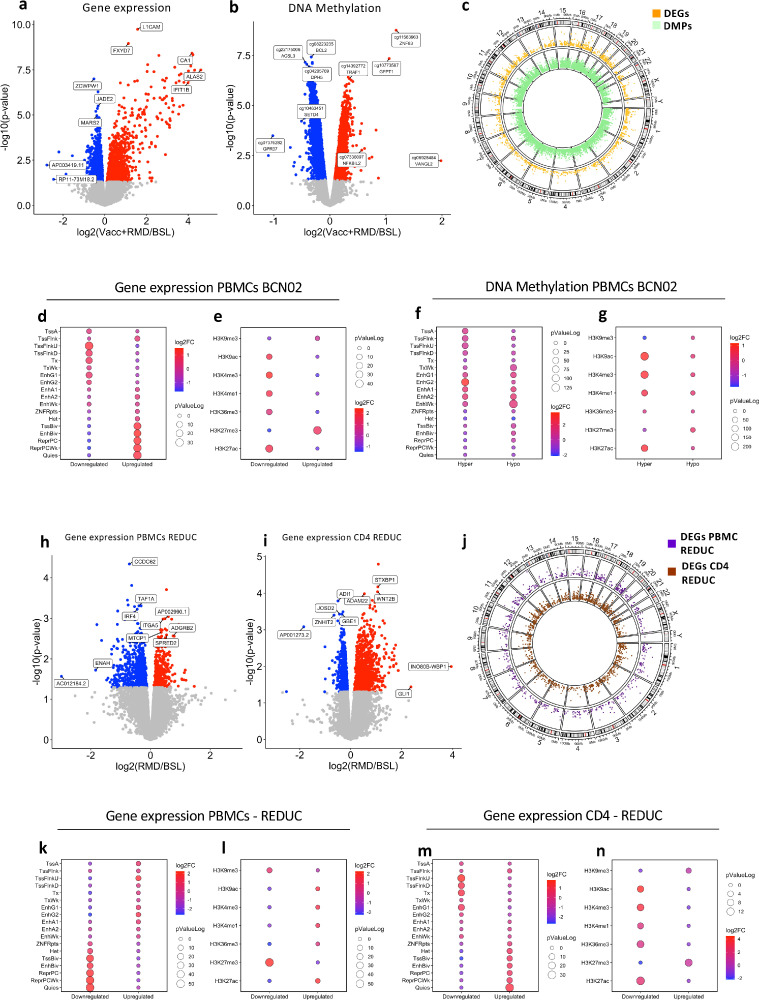
**Effect of combined intervention on gene expression and DNA methylation.** (a) Volcano plot of the 3106 DEGs between time points Vacc+RMD and baseline. (b) Volcano plot of the 15139 DMPs between time points Vacc+RMD and baseline. (c) Manhattan plot in CIRCOS. The outer circle shows the chromosomes and positions of DEGs and DMPs. The inner tracks show the Manhattan plot for DMPs (green) and DEGs (orange) between Vacc+RMD and baseline time points. (d) shows the 18-state ChromHMM enrichment and (e) the histone mark enrichment for DMPs. The same was done for DEGs in (f) and (g). (h) and (i) Volcano plots of for DEGs in total PBMCs and isolated CD4 T cell after RMD-only treatment (REDUC), respectively. Colour key is the same as in (a) and (b). (j) Manhattan plot for RMD-only treatment (REDUC) DEGs in PBMC (purple) and CD4 (brown). (k and m) ChromHMM 18-state enrichment based on DEGs in total PBMCs (in k) or CD4 T cells (in m). Histone marks enrichment for DEGs in PBMC (l) and in CD4 T cells (n). For Volcano plots (a, b, h, i) *X*-axis show the log2 Fold-Change, and *Y*-axis the -log_10_*p*-value. Grey colour indicates *p*-value > 0.05; red, *p*-value > 0.05 and FC > 0 and blue, *p*-value > 0.05 and FC < 0. For ChromHMM and Histone marks enrichments, the colour of the dots is associated with the log2 Fold-change enrichment, and the dot size, with the log_10_*p*-value.

RMD administration impacts the global chromatin condensation state of host genome, thus affecting not only HIV-1 transcription but also other host genes. To evaluate the cumulative effects of the intervention (Vacc+RMD) on the host transcriptome and epigenome, DEGs and DMPs between Vacc+RMD and baseline were identified (Tables S7, 8). Overall, 1404 genes were upregulated and 1561 downregulated (Figure 2a, *p*-value < 0.05, limma model), and after adjusting by false discovery rate 240 genes were upregulated and 103, downregulated considering an adjusted *p*-value < 0.05. Of all DEG (*p*-value < 0.05, limma model), 79% were found in protein coding genes (Table S9). For DNA methylation, there were 7025 hypermethylated and 9114 hypomethylated CpG positions between Vacc+RMD and baseline considering a *p*-value < 0.05 (Figure 2b). After adjusting for multiple comparisons, 1301 DMPs were hypermethylated and 2707 hypomethylated (FDR adjusted *p*-value < 0.05). Of the identified DMPs (*p*-value < 0.05, limma model), 54% were located in regulatory regions (5’UTR, 1stExon, TSS1500 and TSS200), 42%, in body and, 4%, in 3’UTR (Figure S3a, Table S10). The number of DMPs in the regulatory regions 1stExon, TSS1500 and TSS200 were underrepresented compared to their coverage in the array, while the proportion of DMPs in 5’UTR and the body was slightly overrepresented (Table S10). Overall, 30.9% of the DMPs were found in islands, 24.4%, in shores, 7.6%, in shelves, and 37.1% in open sea regions, respectively (Figure S3b, Table S10). The different DEGs and DMPs were distributed in the different chromosomes, and generally, the size of the chromosome was associated with the number of DEGs and DMPs (Figure S3 c, d, Tables S9, 10). Only in chr3, a higher number of DMPs (*p* = 0.01, Chi-square test) was found when compared to their representation in the methylation array (Figure S3d, Tables S9, 10).

Of interest, RMD administration led to the specific upregulation of DNMT3B and EZH2 enzymes (Figure S4), both key upstream regulators of DNA methylation.^36^^,^^37^ Actually, RMD administration, as other HDACi, impact epigenetic cascades at different levels.^38^ To further define potential epigenetic landscape changes from baseline to the Vacc+RMD time points, enrichment analyses were applied on DMPs or DEGs using the ChromHMM and Histone marks in PBMCs from the Roadmap database (E062) (https://egg2.wustl.edu/roadmap/web_portal/chr_state_learning.html) (Figure 2d–g). These ChromHMM enrichment analyses showed that hypermethylated DMPs at Vacc+RMD time point were enriched in regions of active TSS (Transcription start site) and genic enhancers (EnhG2), while hypomethylated DMPs dominated predominantly in regions of weak transcription (Figure 2d). Similarly, downregulated genes were found in regions of active TSS, and upregulated ones, in less active regions or in regions associated with poised expression (Figure 2f). In addition, histone acetylation marks enrichment followed the same trend, with hypermethylated DMPs and downregulated DEGs being enriched in regions of active transcription (e.g.H3K27ac) (Figure 2e and g). These results show that, although RMD is infused to increase histone acetylation, and consequently gene expression, one week after RMD infusion, the majority of upregulated genes were found in regions of transcriptional repression or poised promoters and enhancers rather than regions of active transcription.

Since the Vacc+RMD intervention reflected the cumulative changes induced by MVA.HIVconsv vaccination and RMD infusion (Figure S2g, h), we identified RMD-specific effects, by conducting a transcriptomics assessment on samples from participants in the part A REDUC study (NCT02092116),^6^ in which ART-suppressed participants received 3 cycles RMD without prior vaccination. As in BCN02, the gene expression profiles were compared between baseline and one week after the 3-cycle RMD infusion (post-RMD). Due to limited sample availability, no methylation analyses were conducted in these samples. However total PBMC and isolated CD4 T cell fractions were available for transcriptomic analysis, in which PBMCs showed 224 upregulated and 567 downregulated genes, respectively (Figure 2h, Table S11). When analysing purified CD4 only, a higher number of DEGs was found, 883 upregulated and 613 downregulated (Figure 2i, Table S12). In both sample sets, DEGs were spread among all chromosomes (Figure 2j). Interestingly, the ChromHMM and histone marks enrichment from CD4 DEGs in the REDUC clinical trials (Figure 2k–n) were similar to the results observed in BCN02 (Figure 2f, g).

**Figure 3:**
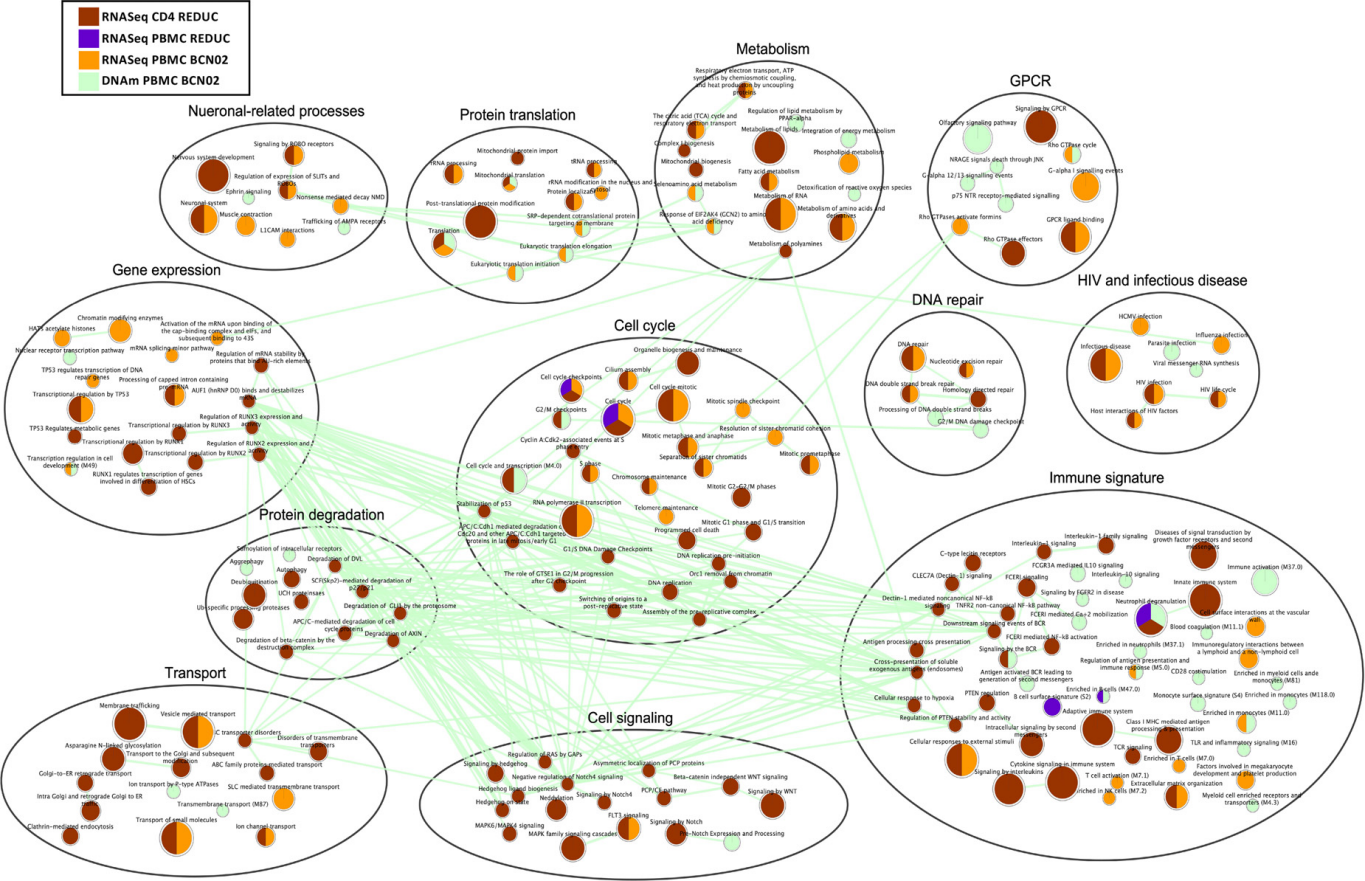
**Effect of RMD on gene expression and DNA methylation in the BCN02 and REDUC trial.** Enrichment Map of enriched pathways and BTMs (GSEA adjusted *p*-value < 0.2) in DEGs before and after RMD infusion in PBMCs of BCN02 (Orange) and in REDUC PBMC (Purple) or isolated REDUC CD4 T cells (Brown). DMPs before and after RMD infusion in BCN02 are shown (Green). The colour of the nodes indicates the different datasets. Edges represent the similarity of the nodes (cutoff = 0.7).

Restoration of dysregulated gene expression profiles and their underlying epigenetic mechanisms may be critical to induce effective antiviral activity in kick-and-kill iinterventions. Therefore, gene set enrichment analyses (GSEA) were conducted to identify the dysregulated pathways upon Vacc+RMD (BCN02) or RMD only treatment (REDUC) (Figure 3, Table S13). For the combined regimen of therapeutic vaccine plus RMD, the data show a strong impact on the HIV pathways in infectious disease and HIV module (*HIV infection* and *HIV life cycle*) as well as on the pathways in immune signature module (e.g. *T cell activation* and *antigen presentation*). Particularly, *Host interactions of HIV factors, HIV infection, HIV life cycle, Infectious Disease* and *Cellular responses to external stimuli*, were enriched in DEGs of REDUC CD4 cells after RMD only and in BCN02 PBMC from individuals undergoing Vacc+RMD (Figure 3). Interestingly, numerous other pathways were shared between the two datasets, including pathways in the metabolism module like *Metabolism of RNA* or *TCA cycle and respiratory electron transport*,) the pathway *Translation* from the protein module, the pathway *Signalling by ROBO receptors* in the module neuronal-related processes, as well as the pathways *DNA double strand break* and *cell cycle mitotic* in modules DNA repair and cell cycle. In parallel, the pathways in modules transport and cell signalling were mainly impacted at transcriptome level in isolated CD4 cells after RMD-only treatment (REDUC). Finally, only few pathways showed an overlap between the two datasets in the module of immune signature, possibly due to the fact that the two studies differ by the inclusion of a therapeutic vaccine in BCN02. Still, among the immune signature module, pathways including *signalling by interleukins, FCERI mediated NF-kB activation* and *antigen processing cross presentation* were dysregulated in isolated CD4 after RMD-only treatment. Interestingly, there was an enrichment in DEGs from PBMC for pathways involving B cell immunity, including *B cell surface* and *Enriched in B cells* and *Antigen activates BCR leading to generation of second messengers* which were observed even in isolated CD4 T cells from the REDUC trial. Of note, the enrichment of the *BCR signalling pathway* in isolated CD4 T includes DEGs also present in the *TCR activation signalling*. A similar observation was made for the *Neuronal-related process* classification, where identified genes are not exclusively associated with processes in the central nervous system but also includes molecules involved in other process, including immunological synapses (GTPases, Calcium Voltage Gated channels, among others). In regards to the T cell related pathways, the majority of them were enriched in DEGs from the BCN02, although *TCR signalling* was also present in isolated CD4 from REDUC (Figure 3). Of note, aside from a large number of pathways in the gene expression module that were modulated by the combination therapy, there was a marked regulation of pathways like *HATs acetylated histones* and *Chromatin modifying enzymes*, indicating the complexity of the epigenetically dysregulated mechanisms upon Vacc+RMD treatment and possibly reflecting RMD activity.

Focusing on the BCN02 dataset only (Figure S5), there were multiple pathways suggesting an epigenetic regulation, as they showed opposite NES. Many of these pathways located to the immune signature module, like *Enriched in Monocytes*, which show a majority of upregulated and hypomethylated genes. Also, *T cells* and *T cell activation*, mainly contained hypermethylated DMPs and downregulated expression of genes. The same trend was observed for *HIV life cycle* pathway and in other pathways of the modules *cell cycle, metabolism, gene expression or protein translation*. Overall these results suggest that after a Vacc+RMD regimen, a homeostatic mechanism might be activated as the global epigenetic state of the cells attempts to counteract the acetylation effects induced by RMD, whose main effects (4 h post infusion) waned 1 week after its administration.

### DNA methylation imprints after intervention can predict viral rebound before ATI

**Figure 4:**
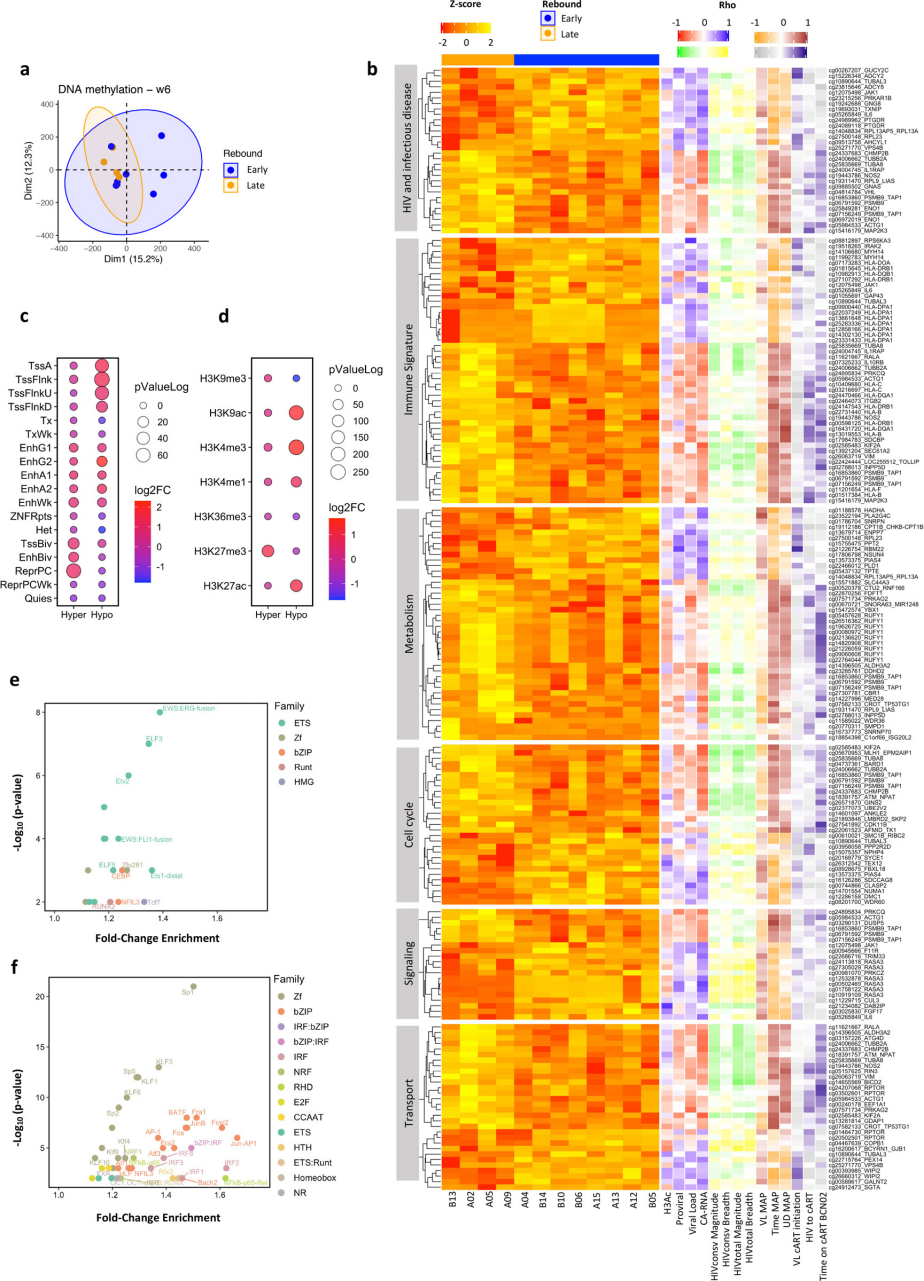
**Differential DNA methylation between individuals with an Early or Late rebound.** (a) PCA for DNA methylation at time point Vacc+RMD. Blue and orange colours indicate Early and Late rebounders, respectively. (b) Heatmap of Z-score methylation levels of DMPs (*p*-value < 0.01) between Early and Late rebound at Vacc+RMD in different pathways (*Y*-axis). The right part of the heatmap shows the correlation (Spearman's Rho) between DMPs methylation levels and different parameters. For correlations with histone 3 acetylation (H3Ac), proviral levels, ultrasensitive viral load and cell-associated RNA (CA-RNA), red colour shows a negative correlation and blue a positive one. For correlations with the magnitude and breadth of the HIVconsv- or HIVtotal-specifc T cell response, green shows a negative correlation and yellow a positive one. For correlations with viral load at day of cART resumption (VL MAP), time on MAP and time in MAP with undetectable viral load (UD MAP), orange shows a negative correlation and brown a positive one. For correlations with VL at cART initiation, time from HIV infection until cART initiation (HIV to cART) and time on cART at BCN02 entry, grey shows a negative correlation and purple a positive one. (c) 18-state ChromHMM enrichment and (d) the histone marks enrichment for DMPs between and Early and Late rebounders. In the *x* axis, *Hyper* and *Hypo* refer to hypermethylated or hypomethylated DMPs in Early rebounding individuals. The Log2 Fold-Change is shown by the dot colour, while the -log_10_*p*-value, with the dot size. (e) Transcription factor (TF) enrichment based on DMPs hypermethylated in Early individuals, and (f) based on DMPs hypomethylated in Early individuals. Colours indicate the TF family, and the name of the TF is shown when Log2 Fold-Change > 0.3. (F) Heatmap of Z-score methylation levels of DMPs (*p*-value < 0.01) between Early and Late rebound at Vacc+RMD in different pathways (*Y*-axis). The right part of the heatmap shows the correlation (Spearman's Rho) between DMPs methylation levels and different parameters. For correlations with histone 3 acetylation (H3Ac), proviral levels, ultrasensitive viral load and cell-associated RNA (CA-RNA), red colour shows a negative correlation and blue a positive one. For correlations with the magnitude and breadth of the HIVconsv- or HIVtotal-specifc T cell response, green shows a negative correlation and yellow a positive one. For correlations with viral load at day of cART resumption (VL MAP), time on MAP and time in MAP with undetectable viral load (UD MAP), orange shows a negative correlation and brown a positive one. For correlations with VL at cART initiation, time from HIV infection until cART initiation (HIV to cART) and time on cART at BCN02 entry, grey shows a negative correlation and purple a positive one.

Next, the hypothesis that changes induced by vaccination and RMD treatment before MAP could explain the different viral rebound kinetics was tested. An unsupervised PCA of DNA methylation data in samples from the Vacc+RMD time point showed individuals with early or late rebound (VL reaching 2000 HIV RNA copies/mL before [“Early rebounders”] or after [“Late rebounders”] 4 weeks of MAP) to cluster closely together (Figure 4a). The same clustering was not observed when performing PCA with gene expression data (Figure S6a). A logistic regression model using the PC1 and PC2 as covariates, confirmed the potential of DNA methylation to discriminate between individuals with an Early versus Late rebound (DNA methylation AUC=0.812 and gene expression AUC=0.594 at the Vacc+RMD time point, Figures 4a and S6a). The heatmaps in Figures 4b and S6b confirm these results. At earlier time points, the discriminating capacity of DNA methylation was lower (AUC BSL = 0.563, AUC Vacc = 0.687), which was also true for gene expression (AUC BSL = 0.563, AUC Vacc = 0.594). Importantly, any clinical parameter, apart from time to viral rebound during MAP, was significantly different between Early and Late rebounders (Table S14).

Given the potential of DNA methylation to discriminate between Early and Late rebounders at the Vacc+RMD time point, the DMPs (*p*-value < 0.05, limma model) between Early and Late rebounders were further analyzed. Of the identified DMPs, 3835 DMPs were hypermethylated and 3761, hypomethylated in Early rebounders; of which 5 and 1 DMPs, respectively, were maintained significantly differentially methylated with a FDR adjusted *p*-value < 0.05 (Figure 4b, Table S15). The hypermethylated DMPs (*p*-value < 0.05, limma model) in Early rebounders were found in bivalent and inactive regions of the genome, which were enriched in the histone mark H3K27me3 (Figure 4c). In contrast, the hypomethylated DMPs in Early rebounders were found in active regions of the genome. Interestingly, when assessing DMPs at transcription factor binding sites (TFBS), hypermethylated DMPs in Early rebounders were strongly enriched for factors of the ETS family, while the hypomethylated DMPs were enriched in transcription factors of the Zf, bZIP and IRF families (Figure 4d, e). These data suggest that the epigenetic landscape after vaccination and RMD infusion may aid in controlling viral replication either directly, by the modulation of chromatin accessibility in regions of active/inactive transcription, or through the binding of specific TFs.

When analysing the main pathways enriched by DMPs between Early and Late rebounders at the Vacc+RMD time point, differential methylation levels were observed in genes involved in pathways and modules related to HIV host factors, immune parameters (*Antigen presentation, TCR signalling* and *signalling by interleukins*), metabolism of lipids and RNA, membrane trafficking and ER-Golgi transport, MAPK and TGF-B signalling, and cell cycle processes (Figure S7). To further characterize these pathways, heatmaps of pathway-associated DMPs (*p*-value < 0.01 and log2FC > |0.5|, limma model) were created and the correlation with viral and immune parameters was studied (Figure 4b and Table S16). These analyses showed that hypomethylated CpGs in Late rebounders and thereby, hypermethylated in Early rebounders, positively correlated with three measures of viral reservoir and replicative activity under ART, including HIV proviral levels, plasma viral load and cell-associate RNA levels (CA-RNA) as well as with the magnitude of the IFNg T cell responses against HIVconsv and HIVtotal (HIV regions not covered by the immunogen) one week before the second vaccination. On the other hand, hypermethylated DMPs in Early individuals were negatively correlated with the time off antiretroviral treatment (time MAP) and the time with undetectable viral load during MAP (UD MAP). In addition, these DMPs showed an association with the time from HIV infection until cART initiation (HIV to cART) as well as the time on cART at BCN02 entry (Time on cART at BCN02). Although DNA methylation levels were not assessed at time points in early and untreated HIV infection, these data suggest that some epigenetic marks could be defined even before ART initiation and that the intrinsic individual epigenome or the impact of the virus during acute infection might define the ability to control HIV-1. This is in line with a recent study that demonstrated certain epigenetic changes induced upon acute HIV-1 infection not to revert upon ART.^39^

Focusing on specific DMPs in the immune signature module (Figures 4b and S8), the methylation levels of *PRKCQ* gene were negatively correlated with cell-associated (CA)-RNA and proviral levels (Figure S8a, h). Interestingly, this gene has been shown to be relevant for HIV-1 latency reversal.^40^^,^^41^ Differential methylation was also observed for genes in the HLA locus. In particular, DNA methylation in positions of the *HLA-DPA1* and *HLA-DRB1* loci were negatively correlated with days off cART parameter (Figure S8b, c, h). In the case of *MAP2K3*, a gene annotated in the modules HIV and immune signature, and hypermethylated in Late rebounders, its methylation levels are negatively correlated with viral load while a positive correlation is observed with the time from HIV infection to cART treatment initiation, the time with undetectable viremia in MAP and the time off cART (Figure S8d, h). On the other hand, in these two categories, the *IL6* and *JAK1* genes were hypomethylated in Late rebounders, with methylation levels positively correlated with CA-RNA. In addition, methylation levels in *IL6* positively correlated with the time of undetectable viral load in MAP, while methylation levels in *JAK1* were significantly related to the magnitude of the T cell response against HIV (Figure S8e, f, h). Next, for the metabolism module there were 9 DMPs associated with the *RUFY1* gene, which was positively correlated to the time on cART at BCN02 entry (Figure S8g, h). Interestingly, the levels of gene associated DMPs classed in metabolism, cell cycle and cell transport with reported interaction with HIV virus (*HADHA*,^42^^,^^43^ *KIF2A*,^44^ *CHMP2B*^44^) correlated with viral and immune parameters (Table S16).

Of special importance, of the 150 DMPs that differed between Early and Late rebounders at the Vacc+RMD time point and represented in the GSEA analysis (Figure S7), 66 were also differentially methylated between the two groups at the baseline time point and maintained a differential methylation signature after vaccination. RMD administration further increased the difference of the median methylation levels between the two groups for 30 of these 66 DMPs. In addition, 52 of the 66 DMPs were also differentially methylated between the two groups in MAP time point. Such results indicate that multiple DMPs between Early and Late rebounders are maintained during MAP and in the presence of viral replication (Table S15).

Together, these results provide evidence that specific host-gene methylation profiles are associated with clinical variables (time on MAP, time on ART, time on HIV), virological parameters (viral loads and HIV proviral levels) and immunological determinations (T-cell responses against HIV and HIVconsv immunogen measured by IFNg ELISpot) and allow for the discrimination between Early and Late rebounders in a kick-and-kill therapeutic vaccination strategy. Additionally, the data also show that RMD impacts the epigenetic landscape broadly and gradually accentuates the early differences in DNA methylation between individuals with an Early or Late virus rebound.

## Discussion

This study assessed the impact of a kick-and-kill strategy combining therapeutic T-cell vaccination and RMD on host transcriptional and epigenomic patterns. It also tested the hypothesis that epigenetic imprints could serve as biomarkers of HIV control during the monitored antiretroviral pause (MAP). BCN02 was a phase I, open-label clinical trial that combined the latency reversing agent (LRA) romidepsin (RMD), an inhibitor of histone deacetylases (HDACi), with HIVconsv vaccination in order to reactivate the HIV provirus at the time of a boosted T-cell response against conserved parts of the virus. During the MAP, 4 out of 13 individuals maintained a VL < 2000 HIV RNA copies/mL for more than 4 weeks.^13^ Systems biology analyses identified molecular pathways, including those involved in infectious diseases and immune activation, that were modulated during the combined intervention at transcriptional and epigenetic levels. Finally, differentially DNA methylated positions (DMPs) after the intervention (Vacc+RMD) discriminated individuals with an Early or Late virus rebound during MAP, with some signatures having been present even before the therapeutic intervention.

The present analysis shows that MVA.HIVconsv vaccination had a marked effect on the expression levels of genes involved in the humoral immune response (e.g. *IGHG1, IGHV1-24 and C1QA*), as well as on genes involved in interleukin signalling. After vaccination, there was also an impact on DNA methylation levels of genes involved in infectious disease pathways (e.g. *APOBEC3G, TUBB3,* and *FURIN*) and, importantly, on genes potentially involved in further downstream epigenetic processes, including *PRC1, MGMT* and *RNF20*. Interestingly, the methylation levels of genes associated with epigenetic processes were directly correlated with the magnitude of the vaccine-elicited T-cell response specific for the conserved HIV regions.^45^, ^46^, ^47^, ^48^, ^49^ This observation is in line with a previous report on influenza vaccination, demonstrating that gene-associated DMPs correlated with the vaccine-induced humoral response.^48^^,^^50^ Similarly, BCG vaccination has been reported to induce an innate immune like-memory in monocytes that was driven by epigenetic processes, and some reports suggest this antigen-unspecific effect may also be induced by other vaccine vectors and adjuvants.^48^^,^^51^^,^^52^ However, in all these analyses, choosing the adequate time point to document possible associations between immune response to vaccination and transcriptomic changes or epigenetic profiles appeared to be critical. This dependence on time point also applies to the present study, were the selection of other time points than the one studied (1 week after the first BCN02 MVA.HIVconsv vaccination) might have helped to capture more profound effects on the vaccine-induced T cell response and allowed to better document the transient changes in transcriptomic activity.^53^ In addition, the observed transcriptomics patterns reflect a recall immune response, as the individuals in BCN02 were previously vaccinated in BCN01 with an heterologous vector prime-boost of ChAd63.HIVconsv and MVA.HIVconsv.^15^ Thus, the selected time points could blur the observation of a major effect on immune transcriptional effects. At the same time, epigenetic signatures, which are more stable over time than transcriptomic changes^54^^,^^55^ may be less sensitive to precise timing.

The availability of samples from the REDUC trial, where participants received RMD without a therapeutic T-cell vaccine, allowed to assess the contribution of RMD to the combined intervention in BCN02. Indeed, this data show that RMD administration alone had a marked effect on both, gene expression and DNA methylation, although at gene-level, no major negative correlations were captured among them. This outcome is likely partially due to the low sample size and the different time to respond to stimuli. Nevertheless, different DMPs and DEGs converged in the same pathways, suggesting an epigenetic regulation of these pathways. Among the pathways modulated by Vacc+RMD, pathways related to HIV life cycle and infectious diseases were identified as the most severely impacted. The fact that these observations were made using samples taken after the most prominent effect of RMD on histone acetylation had already waned, suggests that a dysregulation of HIV transcription is further maintained*.* In fact, the REDUC trial showed that while RMD was able to induce the initiation and elongation of the virus transcription, the drug failed to increase late events in HIV-1 transcription.^56^ This finding is in line with several other reports that suggest that more potent LRA, and potentially combinations of them would be needed to efficiently purge the viral reservoir. Combining LRA could indeed allow for broader reactivation of latent virus through activity on different distinct integration sites in the host genome and in different cell types.^57^^,^^58^

In addition to a strong latency reversing activity that is able to induce HIV antigen expression, the stimulation of an effective immune response to eliminate cells with reactivated virus will also be critical to achieve HIV-1 remission in the absence of ART.^8^^,^^59^ In the BCN02 study, the virus-specific T-cell immunity was clearly enhanced during the therapy^13^^,^^60^ and was found here to relate to pathways (including T-cell activation, TLR signalling and/or antigen presentation) that were modulated at both, the epigenetic and transcriptional levels. These pathways were also observed in the transcriptional program of CD4 cells from the individuals in the REDUC clinical trial, in which only RMD was administered. These findings suggest that the vaccination of individuals in BCN01 and BCN02 prior to RMD infusion may have poised the immune system to respond and exert its antiviral activity against HIV. Indeed, *in vitro* studies showed that inducing an efficient HIV-specific CD8 response before the administration of a LRA resulted in a more effective removal of infected cells.^8^

Genes in pathways of HIV infection, immune signature and metabolism categories, among others, showed a differential DNA methylation between individuals with an Early and Late viral rebound. Interestingly, some of these methylation imprints were already observed at baseline could thus be host-specific baseline characteristics or, alternatively, be modulated by HIV infection, antiretroviral treatment or previous prime-boost vaccination in BCN01. Thus, the present data hint at the possibility that epigenetic signatures, including those assessed before kick-and-kill interventions, could predict eventual virus control in treatment interruption. Furthermore, a recent study has associated specific DNA methylation marks with the size of the viral reservoir and the time to viral rebound during an ATI in a clinical trial administrating a latent reactivation agent (NCT01680094).^61^ The potential of epigenome studies in the identification of correlates of control of HIV-1 infection is also in line with a previous report from our group showing that differential capacity to control HIV infection in the absence of ART was associated with a differential methylation profile of genes involved in antiviral response and T-cell immunity.^16^^,^^62^ Finally, different DNA methylation in CD4 T cells has been associated with distinct viral progression and can be impacted by cART treatment initiation,^17^ again suggesting that epigenetic profiling, even in early stages of HIV infection, may have predictive power for post-treatment control.

Previous report in Cutaneous T Cell Lymphoma showed that RMD increased the chromatin accessibility mainly of regions with open or relaxed chromatin,^63^ while the present data showed that RMD administration further potentiated the epigenetic differences between individuals with an early or late viral rebound. Such observations were not present at transcriptional level (Figure S4B, C). Interestingly, at Vacc+RMD time point, Early rebounders showed hypomethylation mainly in regions of active transcription, while Late rebounders were hypomethylated in regions of repressed chromatin. This differential epigenetic landscape could also affect viral rebound, since late rebounding individuals may have HIV-1 integrated in regions of the host genome that are transcriptionally less active, as has been suggested in the setting of natural HIV control.^64^ In this line, effects on DNA methylation were observed in CpG positions of a common integration site, *BACH2*^65^ (Figure S9). Specifically, the cg03035849_BACH2 site was hypermethylated in Early rebounders compared with Late rebounders one week after RMD administration and positively correlated with CA-RNA. Similarly, two recently described integration sites, *RASA3* and *RPTOR*,^66^, ^67^, ^68^ were differentially methylated between Early and Late rebounders after romidepsin administration, and also correlated with proviral and CA-RNA levels (Figure S9), thus further suggesting that the host´s epigenetic landscape may be a critical determinant for viral integration.

The limitations of this work include small study size, limited sampling time points and lack of a placebo control arm. This prevented us from determining, for example, to what extent the basal levels of DNA methylation (BCN01 enrolment) could have predicted viral rebound kinetics during the treatment interruption phase. Actually, in well powered studied selection of a combination of DNA methylation candidates and viral and clinical data may yield with predictive models of viral rebound. In addition, the coverage of the methylation studies could be increased by using the EPIC Beadchip array rather than the 450K Beadchip array, as the former includes additional DMPs on regulatory elements that may be relevant to understand the differential viral rebound. Another limitation is the unbalanced sex distribution in this study with only one female participant. This prevented us from applying statistical correction methods and, given the distinct clustering of this individual, obliged us to exclude this participant from the omics analyses. Furthermore, and although HIVconsv was designed to elicit T-cell responses, the gene expression signature one week after vaccination included markers of immunoglobulins, Fc receptors and complement activation. This suggests that vaccination may have driven some humoral immune activation, including some that could target the viral vector MVA. Unfortunately, no B cell immune-monitoring was included in BCN02, although this should be considered in future clinical interventions. Another potential limitation was the study of total PBMCs rather than sorted lymphocyte subsets, as it is not possible to determine the precise cellular origin of some of the observed signals. To overcome this limitation, samples from the REDUC trial were evaluated and showed different signals from PBMC or from isolated CD4 T cells. Interestingly, the transcriptional patterns in the isolated CD4+ T cells were similar to those observed in PBMCs from the BCN02 study. With this result, it is tempting to speculate that previous vaccination may increase the signals from CD4 T cells in BCN02 clinical trial, although this should be tested in CD4 T cells isolated fractions from BCN02 study, which are unfortunately unavailable. Therefore, although from a biomarker point of view, the use of total PBMCs is potentially still appealing, for the identification of possible therapeutic targets, isolated cell types studies might be more informative.

Together, this is a unique exploratory study of a clinical trial testing a kick-and-kill strategy that showed a partial clinical signal in terms of virus control during treatment interruption. The data clarified the different molecular pathways that were modulated in response to this specific intervention and highlighted that DNA methylation profiling could be further explored as a biomarker to predict viral control before treatment interruption. These results warrant further confirmation in larger, placebo-controlled clinical trials and may inform future refined strategies to achieve a functional HIV cure.

## Contributors

MRR and CB designed the experimental plan. BM, JM and CB contributed with clinical trial, patient data and sample management. BCl, BM, JM, OS, MM, were in charge of patient recruitment and sample provision. BOT, CDC, SC and ALL did the sample processing. BOT, AEC and EG performed the transcriptome analysis and BOT, MRR and FCM analyzed methylome data and their integration with clinical and virological parameters and immune reactivity data. ME and MB performed DNA Methylation assays and helped in data analysis. BM, ALL, MRU and SC assessed HIV specific T cell responses. MCP and JMP determined CA-HIV-1 DNA, CA-HIV-1 RNA and residual viral load. BOT conducted R language and programing for the execution of statistically, bioinformatic and integrative analyses with support of AEC, AS and MLC. BOT, MRR, and CB did the results interpretation. BOT, MRR, CB, BC, BM, MT, TH, DH, and RP participate in scientific discussions. BOT, CB and MRR drafted and edited the manuscript together. All authors reviewed and approved the final version of the manuscript.

### Data sharing statement

All gene expression and DNA methylation data were uploaded in GEO under accession numbers: GSE184653, GSE185391 and GSE185027, which include sample basic information including sample source, age, sex, time point and viral rebound. Additional information can be found in the supplementary material of this article. Additionally, the R code applied in the analyses of this article can be found in the GitHub link: https://github.com/hostimmuneOMICS/BCN02_OmicsAnalysis.

## Declaration of interests

BM is a consultant of AELIX THERAPEUTICS, S.L outside the submitted work. CB is co-founder, chief science officer and shareholder of AELIX THERAPEUTICS. J.M. has received research funding, consultancy fees and lecture sponsorships from and has served on advisory boards for various companies (MSD, Gilead Sciences, Viiv Healthcare, and Janssen-Cilag). All other authors declare that they have no competing interests.

## Appendix Supplementary materials

Image, application 1

Image, application 2

Image, application 3

Image, application 4

Image, application 5

Image, application 6

Image, application 7

Image, application 8

Image, application 9

Image, application 10
