# Supplementary material for: Epigenetic landscape in the kick-and-kill therapeutic vaccine BCN02 clinical trial is associated with antiretroviral treatment interruption (ATI) outcome
Source: eBioMedicine. 2022 Mar 21;78:103956. doi: 10.1016/j.ebiom.2022.103956 (PMC8938861; doi:10.1016/j.ebiom.2022.103956)
Supplement: Supplementary file 1 [file mmc1.pdf]

Figure S1

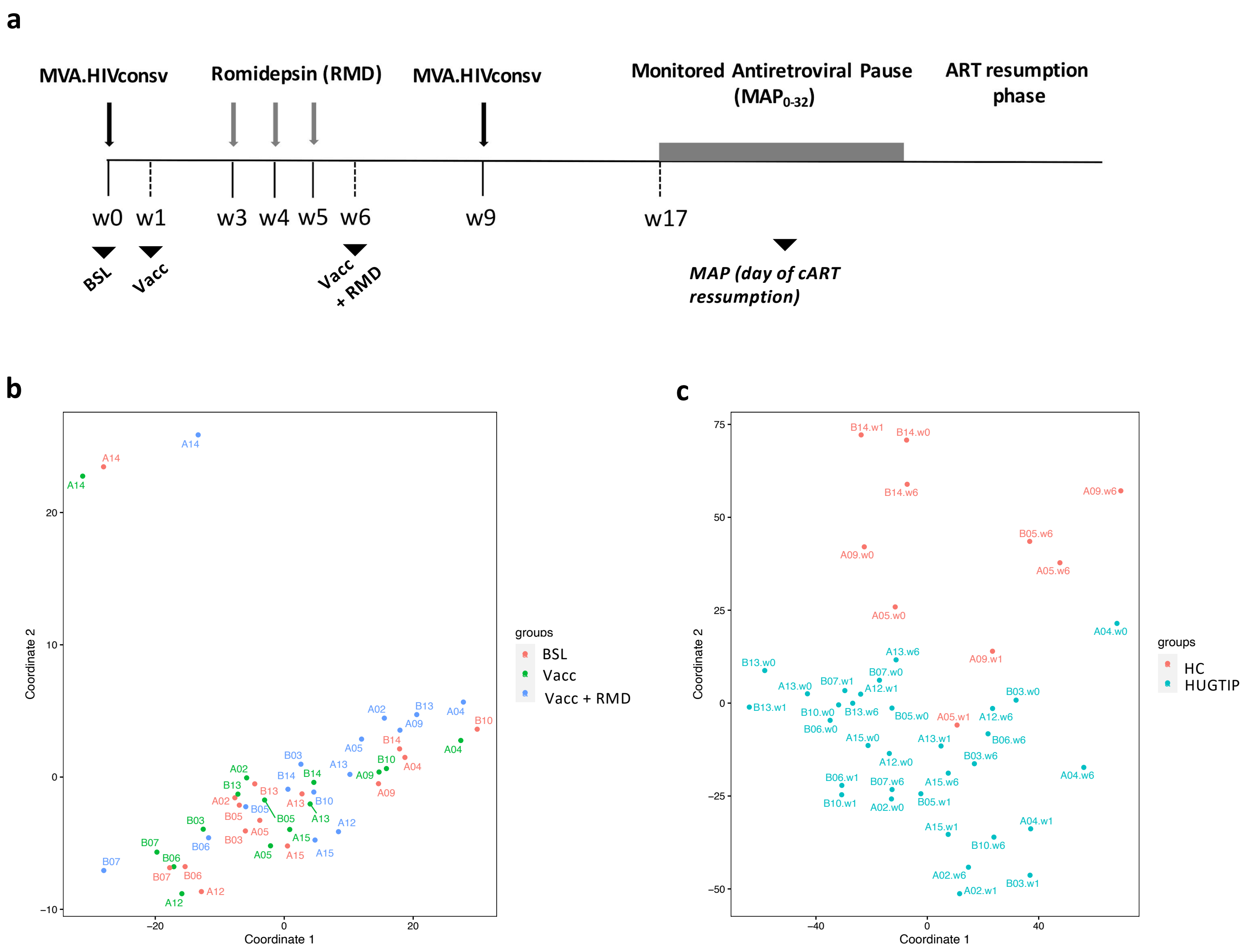
a
MVA.HIVconsv
Romidepsin (RMD)
MVA.HIVconsv
Monitored Antiretroviral Pause (MAP0-32)
ART resumption phase
w0 w1 w3 w4 w5 w6 w9 w17
BSL
Vacc
Vacc + RMD
MAP (day of cART ressumption)
b
Coordinate 1
Coordinate 2
groups
BSL
Vacc
Vacc + RMD
c
Coordinate 1
Coordinate 2
groups
HC
HUGTIP

Figure S2

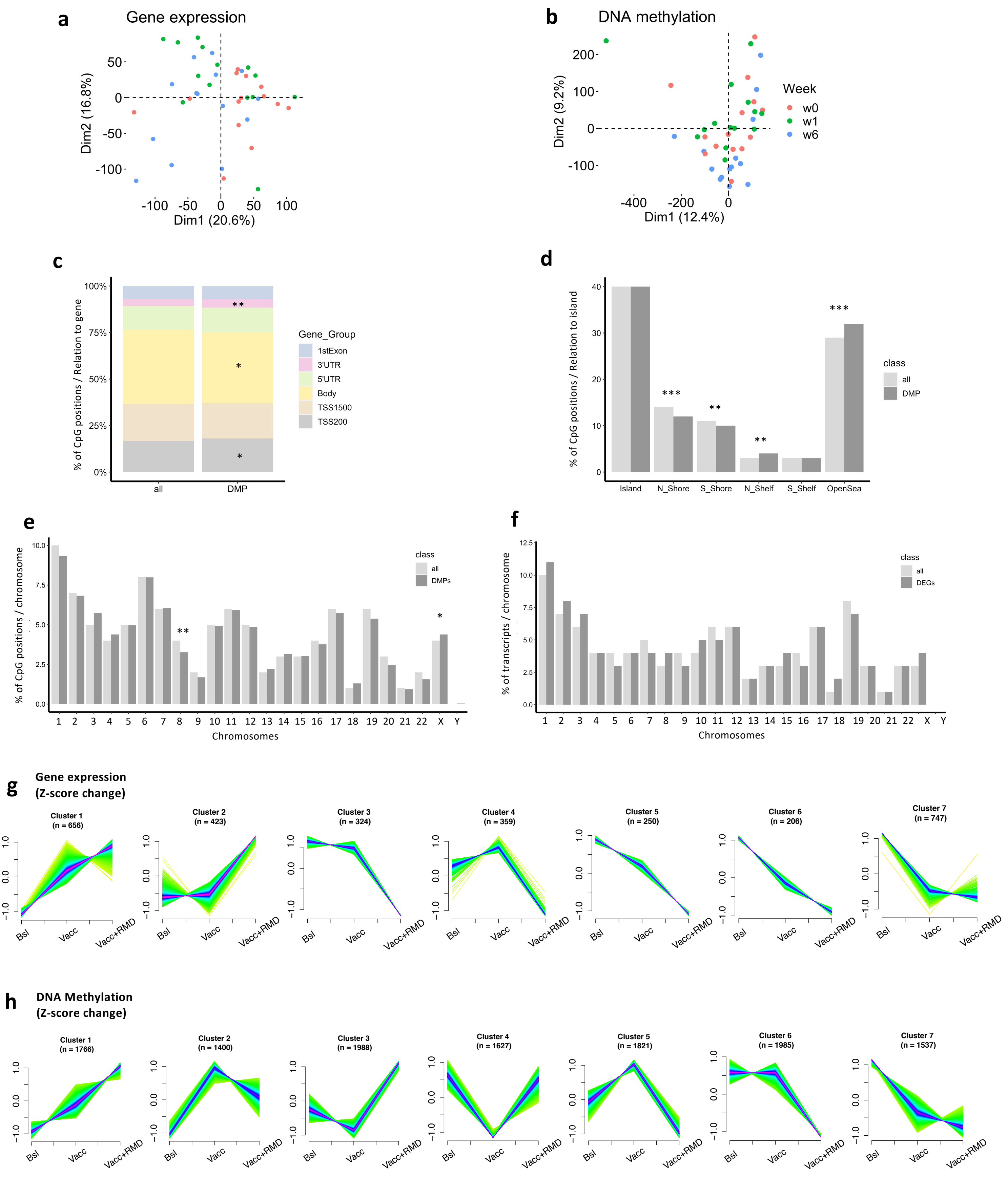

a
Gene expression
Dim2 (16.8%)
Dim1 (20.6%)
b
DNA methylation
Dim2 (9.2%)
Dim1 (12.4%)
Week
w0
w1
w6
c
% of CpG positions / Relation to gene
Gene_Group
1stExon
3'UTR
5'UTR
Body
TSS1500
TSS200
all
DMP
d
% of CpG positions / Relation to island
class
all
DMP
Island
N_Shore
S_Shore
N_Shelf
S_Shelf
OpenSea
e
% of CpG positions / chromosome
class
all
DMPs
Chromosomes
f
% of transcripts / chromosome
class
all
DEGs
Chromosomes
g
Gene expression
(Z-score change)
Cluster 1 (n = 656)
Cluster 2 (n = 423)
Cluster 3 (n = 324)
Cluster 4 (n = 359)
Cluster 5 (n = 250)
Cluster 6 (n = 206)
Cluster 7 (n = 747)
Bsl
Vacc
Vacc+RMD
h
DNA Methylation
(Z-score change)
Cluster 1 (n = 1766)
Cluster 2 (n = 1400)
Cluster 3 (n = 1988)
Cluster 4 (n = 1627)
Cluster 5 (n = 1821)
Cluster 6 (n = 1985)
Cluster 7 (n = 1537)
Bsl
Vacc
Vacc+RMD

Figure S3

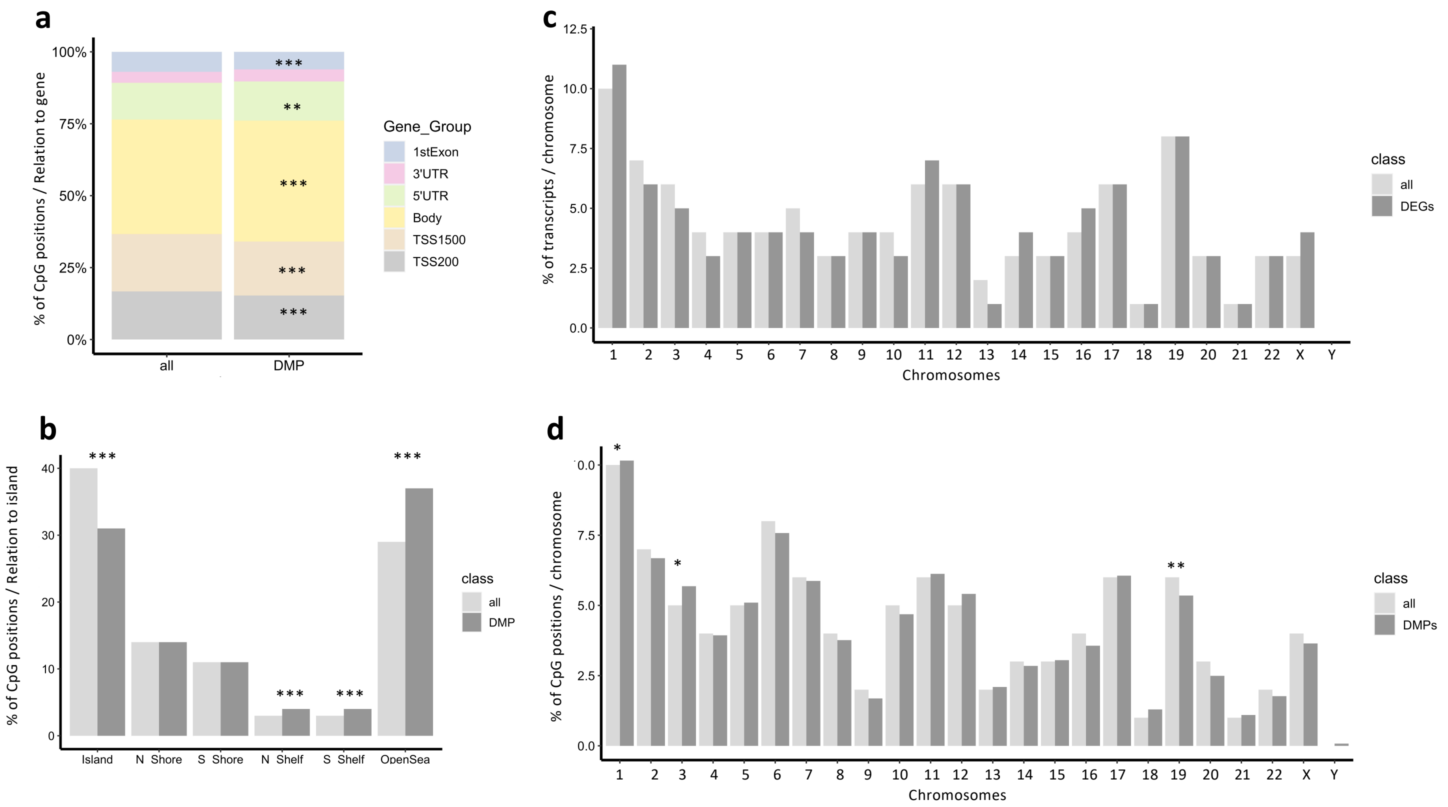

a
% of CpG positions / Relation to gene
100%
75%
50%
25%
0%
all
DMP
***
**
***
***
***
Gene_Group
1stExon
3'UTR
5'UTR
Body
TSS1500
TSS200
b
% of CpG positions / Relation to island
40
30
20
10
0
Island
N_Shore
S_Shore
N_Shelf
S_Shelf
OpenSea
class
all
DMP
c
% of transcripts / chromosome
12.5
10.0
7.5
5.0
2.5
0.0
1 2 3 4 5 6 7 8 9 10 11 12 13 14 15 16 17 18 19 20 21 22 X Y
Chromosomes
class
all
DEGs
d
% of CpG positions / chromosome
7.5
5.0
2.5
0.0
1 2 3 4 5 6 7 8 9 10 11 12 13 14 15 16 17 18 19 20 21 22 X Y
Chromosomes
class
all
DMPs

Figure S4

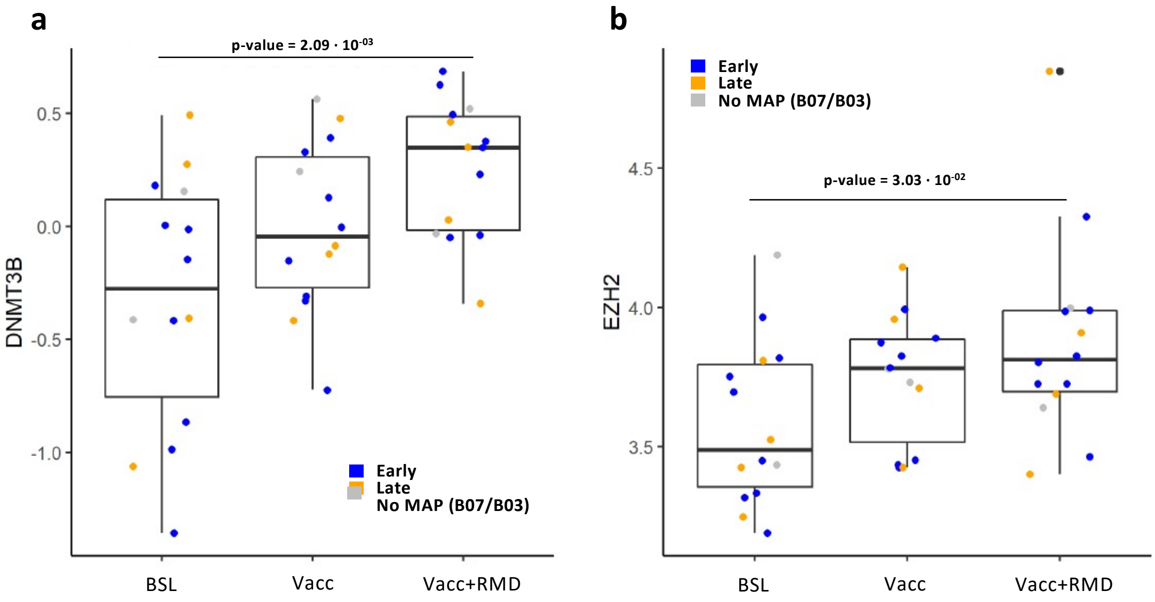
a
p-value = 2.09 · 10-03
DNMT3B
0.5
0.0
-0.5
-1.0
Early
Late
No MAP (B07/B03)
BSL
Vacc
Vacc+RMD
b
Early
Late
No MAP (B07/B03)
p-value = 3.03 · 10-02
EZH2
4.5
4.0
3.5
BSL
Vacc
Vacc+RMD

Figure S5

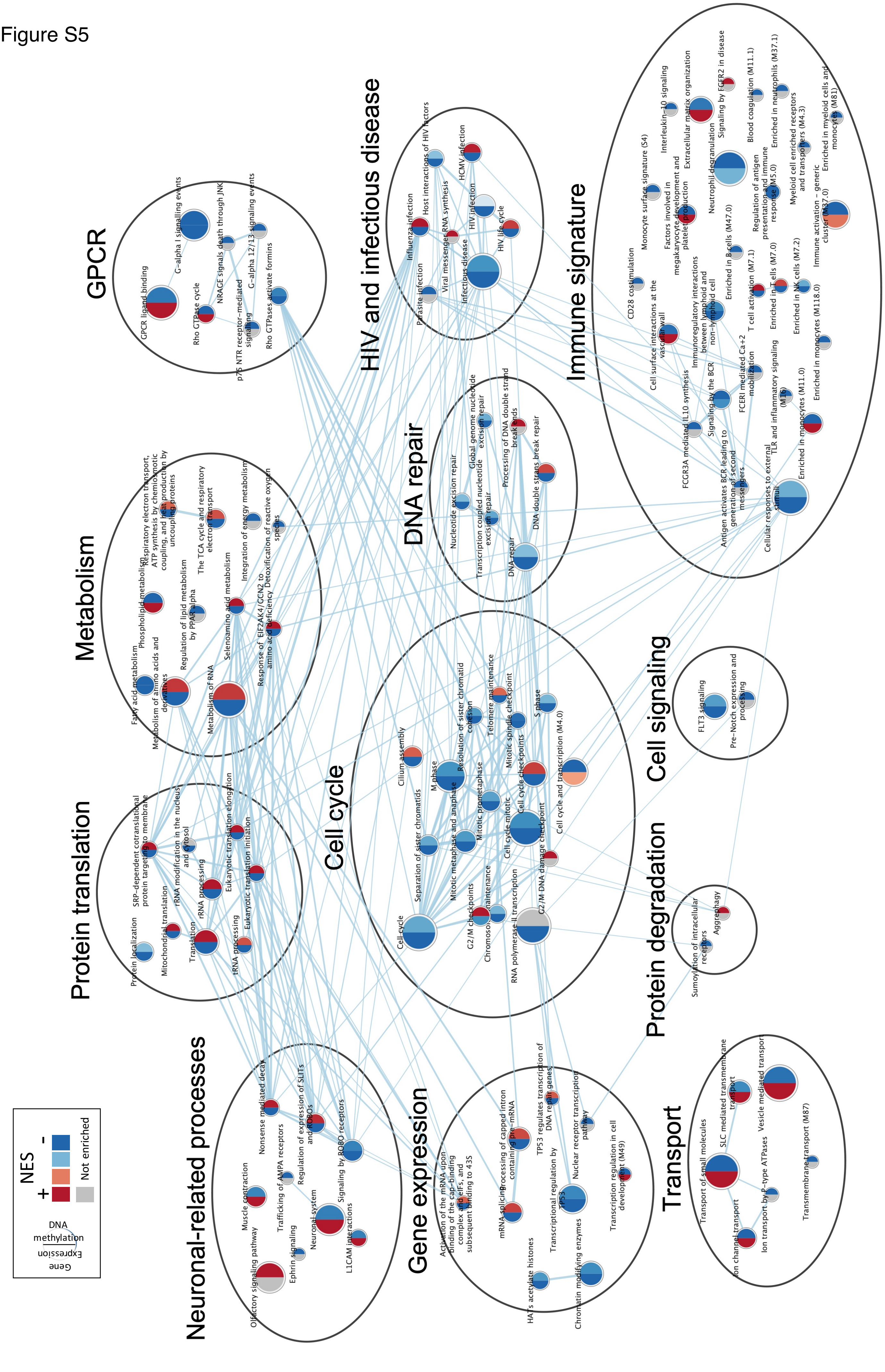
GPCR
GPCR ligand binding
G-alpha I signalling events
NRAGE signals death through JNK
G-alpha 12/13 signalling events
Rho GTPase cycle
p75 NTR receptor-mediated signalling
Rho GTPases activate formins
HIV and infectious disease
Host interactions of HIV factors
HCMV infection
Influenza infection
Viral messenger RNA synthesis
HIV infection
HIV life cycle
Parasite infection
Infectious disease
Immune signature
Interleukin-10 signaling
Extracellular matrix organization
Signaling by FCER2 in disease
Blood coagulation (M11.1)
Enriched in neutrophils (M37.1)
Monocyte surface signature (S4)
Factors involved in megakaryocyte development and platelet production
Neutrophil degranulation
Regulation of antigen presentation and immune response (M5.0)
Myeloid cell enriched receptors and transporters (M4.3)
Enriched in myeloid cells and monocytes (M81)
Immune activation - generic cluster (M37.0)
CD28 costimulation
Enriched in B cells (M47.0)
Cell surface interactions at the vascular wall
Immunoregulatory interactions between lymphoid and non-lymphoid cell
T cell activation (M7.1)
Enriched in T cells (M7.0)
Enriched in NK cells (M7.2)
Enriched in monocytes (M118.0)
FCGR3A mediated IL10 synthesis
Signaling by the BCR
FCERI mediated Ca+2 mobilization
TLR and inflammatory signaling (M16)
Enriched in monocytes (M11.0)
Antigen activates BCR leading to generation of second messengers
Cellular responses to external stimuli
DNA repair
Global genome nucleotide excision repair
Processing of DNA double strand break ends
Nucleotide excision repair
Transcription coupled nucleotide excision repair
DNA double strand break repair
DNA repair
Metabolism
Respiratory electron transport, ATP synthesis by chemiosmotic coupling, and heat production by uncoupling proteins
The TCA cycle and respiratory electron transport
Integration of energy metabolism
Detoxification of reactive oxygen species
Phospholipid metabolism
Regulation of lipid metabolism by PPARalpha
Selenoamino acid metabolism
Response of EIF2AK4/GCN2 to amino acid deficiency
Fatty acid metabolism
Metabolism of amino acids and derivatives
Metabolism of RNA
Cell cycle
Cilium assembly
M phase
Resolution of sister chromatid cohesion
Telomere maintenance
Mitotic spindle checkpoint
S phase
Cell cycle and transcription (M4.0)
Cell cycle checkpoints
Mitotic prometaphase
Separation of sister chromatids
Mitotic metaphase and anaphase
Cell cycle mitotic
G2/M DNA damage checkpoint
Cell cycle
G2/M checkpoints
Chromosome maintenance
RNA polymerase II transcription
Cell signaling
FLT3 signaling
Pre-Notch expression and processing
Protein translation
SRP-dependent cotranslational protein targeting to membrane
rRNA modification in the nucleus and cytosol
Eukaryotic translation elongation
Eukaryotic translation initiation
Mitochondrial translation
rRNA processing
Protein localization
Translation
tRNA processing
Protein degradation
Aggrephagy
Sumoylation of intracellular receptors
Neuronal-related processes
Nonsense mediated decay
Regulation of expression of SLITs and ROBOs
Signaling by ROBO receptors
Muscle contraction
Trafficking of AMPA receptors
Neuronal system
Ephrin signaling
L1CAM interactions
Olfactory signaling pathway
Gene expression
Activation of the mRNA upon binding of the cap-binding complex and eIFs, and subsequent binding to 43S
Processing of capped intron containing pre-mRNA
TP53 regulates transcription of DNA repair genes
Nuclear receptor transcription pathway
Transcription regulation in cell development (M49)
mRNA splicing
Transcriptional regulation by TP53
HATs acetylate histones
Chromatin modifying enzymes
Transport
SLC mediated transmembrane transport
Vesicle mediated transport
Transport of small molecules
Ion transport by P-type ATPases
Transmembrane transport (M87)
Ion channel transport
NES
+
-
Not enriched
DNA methylation
Gene Expression

Figure S6

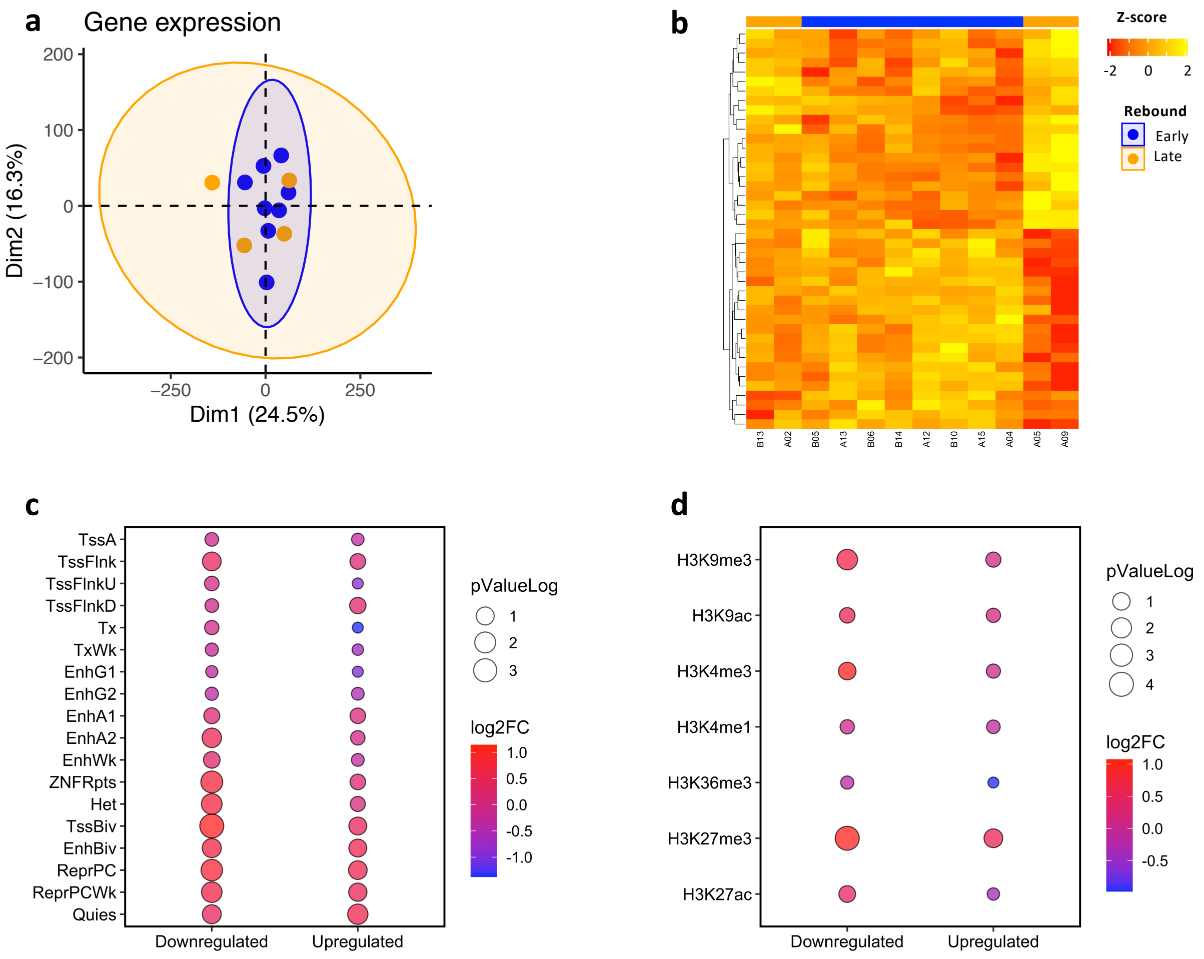
a
Gene expression
Dim1 (24.5%)
Dim2 (16.3%)
200
100
0
−100
−200
−250
0
250
b
Z-score
-2
0
2
Rebound
Early
Late
B13
A02
B05
A13
B06
B14
A12
B10
A15
A04
A05
A09
c
TssA
TssFlnk
TssFlnkU
TssFlnkD
Tx
TxWk
EnhG1
EnhG2
EnhA1
EnhA2
EnhWk
ZNFRpts
Het
TssBiv
EnhBiv
ReprPC
ReprPCWk
Quies
Downregulated
Upregulated
pValueLog
1
2
3
log2FC
1.0
0.5
0.0
-0.5
-1.0
d
H3K9me3
H3K9ac
H3K4me3
H3K4me1
H3K36me3
H3K27me3
H3K27ac
Downregulated
Upregulated
pValueLog
1
2
3
4
log2FC
1.0
0.5
0.0
-0.5

Figure S7

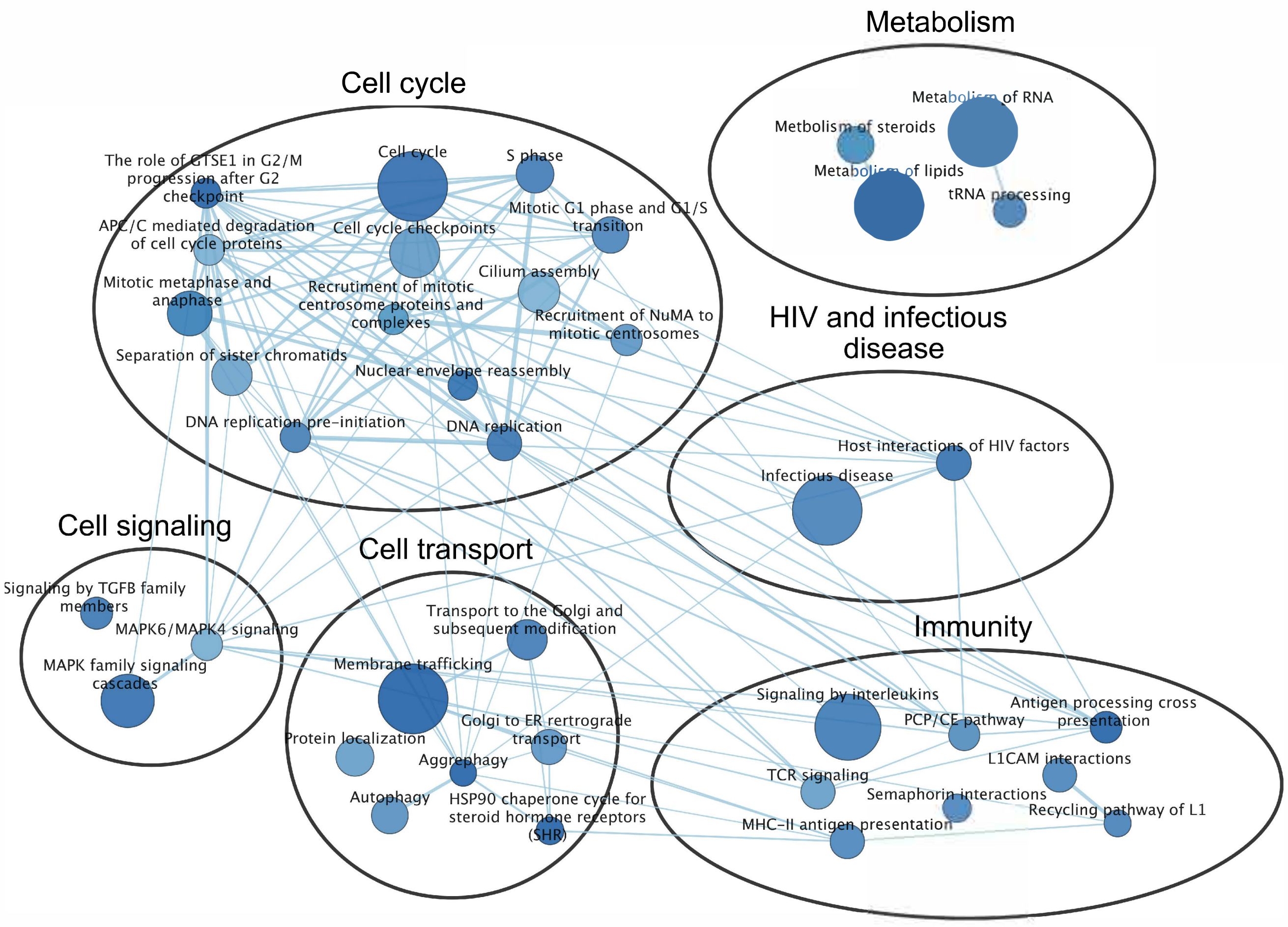
Metabolism
Metabolism of RNA
Metbolism of steroids
Metabolism of lipids
tRNA processing
Cell cycle
The role of GTSE1 in G2/M progression after G2 checkpoint
Cell cycle
S phase
APC/C mediated degradation of cell cycle proteins
Cell cycle checkpoints
Mitotic G1 phase and G1/S transition
Cilium assembly
Mitotic metaphase and anaphase
Recrutiment of mitotic centrosome proteins and complexes
Recruitment of NuMA to mitotic centrosomes
Separation of sister chromatids
Nuclear envelope reassembly
DNA replication pre-initiation
DNA replication
HIV and infectious disease
Host interactions of HIV factors
Infectious disease
Cell signaling
Signaling by TGFB family members
MAPK6/MAPK4 signaling
MAPK family signaling cascades
Cell transport
Transport to the Golgi and subsequent modification
Membrane trafficking
Golgi to ER rertrograde transport
Protein localization
Aggrephagy
Autophagy
HSP90 chaperone cycle for steroid hormone receptors (SHR)
Immunity
Signaling by interleukins
PCP/CE pathway
Antigen processing cross presentation
L1CAM interactions
TCR signaling
Semaphorin interactions
Recycling pathway of L1
MHC-II antigen presentation

Figure S8

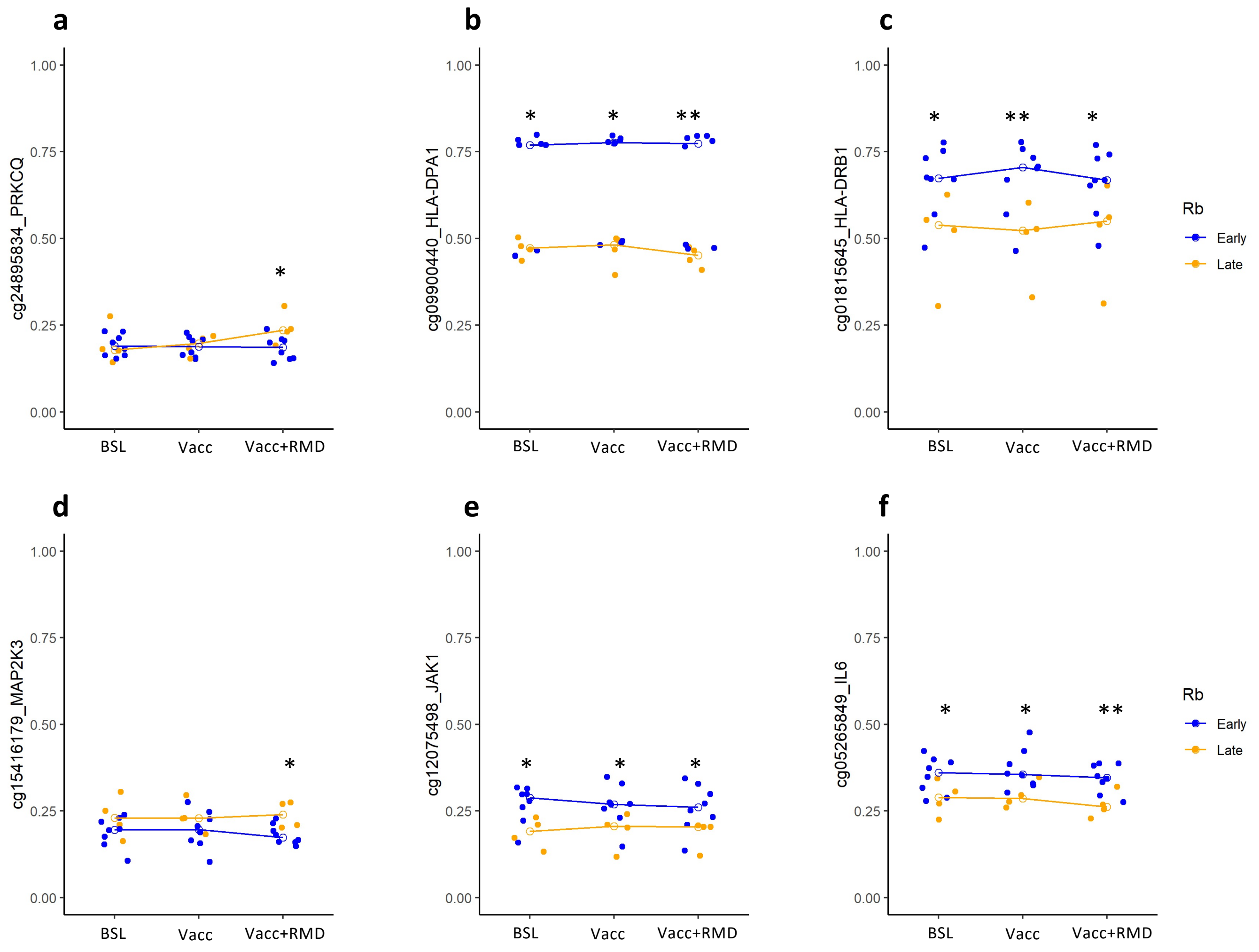

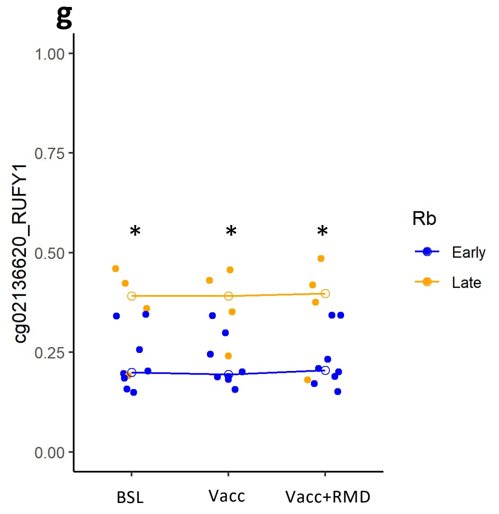

h

	Variable	Rho	p-value
**cg24895834_PRKCQ**	CA-RNA	-0.56	$3.77 \cdot 10^{-0.2}$
**cg09900440_HLA-DPA1**	CA-RNA	0.53	$4.99 \cdot 10^{-0.2}$
**cg09900440_HLA-DPA1**	Viral Load	0.57	$3.09 \cdot 10^{-0.2}$
**cg09900440_HLA-DPA1**	Days off cART	-0.72	$7.59 \cdot 10^{-0.3}$
**cg01815645_HLA-DRB1**	Viral Load	0.57	$3.25 \cdot 10^{-0.2}$
**cg01815645_HLA-DRB1**	Days off cART	-0.84	$6.64 \cdot 10^{-0.5}$
**cg15416179_MAP2K3**	Viral Load	-0.62	$1.89 \cdot 10^{-0.2}$
**cg15416179_MAP2K3**	Days off cART	0.58	$4.92 \cdot 10^{-0.2}$
**cg05265849_IL6**	CA-RNA	0.67	$9.22 \cdot 10^{-0.3}$
**cg12075498_JAK1**	CA-RNA	0.67	$8.31 \cdot 10^{-0.3}$
**cg02136620_RUFY1**	Time on cART at BCN02 entry	0.69	$1.30 \cdot 10^{-0.2}$
**cg02136620_RUFY1**	Undetectable MAP	0.59	$4.41 \cdot 10^{-0.2}$

Figure S9

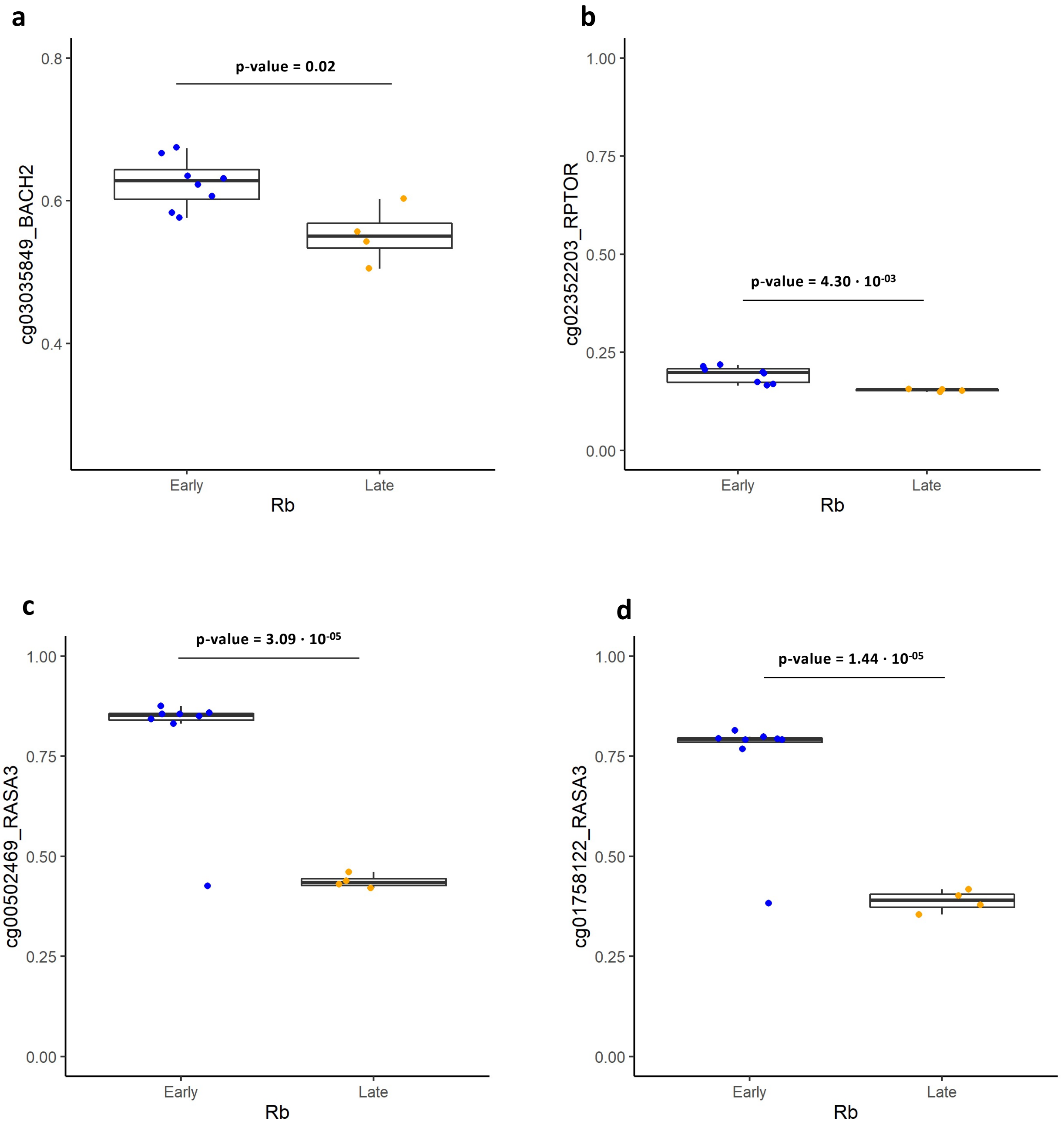

e

	Variable	Rho	p-value
**cg03035849_BACH2**	CA-RNA	0.59	0.03
**cg02352203_RPTOR**	CA-RNA	0.59	0.026
**cg00502469_RASA3**	Proviral	0.54	0.048
**cg00502469_RASA3**	CA-RNA	0.72	0.003
**cg01758122_RASA3**	CA-RNA	0.70	0.005
