# Supplementary material for: Epigenetic landscape in the kick-and-kill therapeutic vaccine BCN02 clinical trial is associated with antiretroviral treatment interruption (ATI) outcome
Source: eBioMedicine. 2022 Mar 21;78:103956. doi: 10.1016/j.ebiom.2022.103956 (PMC8938861; doi:10.1016/j.ebiom.2022.103956)
Supplement: Supplementary file 10 [file mmc10.docx]

**Figure S1. BCN02 Scheme and exploratory analysis** (a) Scheme of the BCN02 clinical trial. (b) MDS plot of DNA methylation. Each of the points represents one sample, colored according to the sampling time point. The plot shows samples from A14, the only female participant in the study, to be in a different cluster. (c) MDS plot of gene expression after removal of participant A14. Each of the points represents one sample, colored according to the laboratory where the sample was processed.

**Figure S2. Impact of vaccination in gene expression and DNA methylation changes and longitudinal profiles along the intervention**. (a) and (b) show PCA according to normalized gene expression and DNA methylation (colors according to sampling time points). (c) Percentage of CpGs according to relation to genes in DMPs (Vacc-BSL) and in 450K array (All). (d) Percentage of CpGs according to relation to Island in DMPs (Vacc-BSL) and in 450K array (All). (e) shows the proportion of genes in each chromosome in DMPs (Vacc-BSL) and in 450K array. (f) Percentage of genes in each chromosome in DEGs (Vacc-BSL) and all the genes that passed the QC. * indicates p-value < 0.05, ** p-value < 0.01 and *** p-value < 0.001(chi-squared test). g) and h) show the longitudinal gene expression (g) and DNA methylation (h) cluster profiles during the clinical trial based on standardized data. Color indicates the membership value, that indicated how well a gene fits to the profile. While green indicated low membership value, pink indicates high membership value.

**Figure S3. DMPs and DEGs between the Vacc+RMD and the baseline time point.** (a) Proportion of

CpGs according to their relation to genes in the DMPs and in 450K array (All). (b) Percentage of CpGs according to their relation to Island in DMPs and in 450K array (All). (c) and (d) proportions of genes in each chromosome in DMPs and 450K array (All) and DEGs and all the genes that passed the QC (All), respectively. * indicates p-value < 0.05, ** p-value < 0.01 and *** p-value < 0.001 (chi-squared test).

**Figure S4. Gene expression of DNMT3B and EZH2 at baseline, Vacc, and Vacc+RMD time points.** (a) and (b) show gene expression levels for DNMT3B and EZH2, respectively. Gene expression is shown as Log2 normalized (TMM method) counts. The expression of these two genes is increased at Vacc+RMD in contrast to baseline. * indicates p-value < 0.05, ** p-value < 0.01 and *** p-value < 0.001 (limma model). The colour of the points indicate Early rebounders (blue), Late rebounders (orange) or the individuals not considered in comparisons between Early and Late individuals (grey).

**Figure S5. Differentially expressed genes and differentially methylated positions in the BCN02 study**

**between the Vacc+RMD and baseline time points.** Pathways and BTMs enrichment in DMPs and

DEGs (GSEA result with adjusted p-value < 0.2 in the enrichment for gene expression or DNA methylation,

Table S13 show the raw results). The different nodes are divided into two parts, the left part refers to gene

expression, and the right part to DNA methylation. Red color indicates a positive NES (Normalized

Enrichment Score), and blue color, a negative NES. Grey color indicated that no enrichment was observed. Edges represent the similarity of the nodes (cutoff = 0.4).

**Figure S6. DEGs between Early and Late individuals at the Vacc+RMD time points.** (a) PCAs for gene

expression at time point Vacc+RMD colored according to Early (Blue) or Late rebound (Orange). (b) Heatmap of DEGs (p-value < 0.01, limma model, n = 42) between Early (Blue) and Late (Orange) rebounders at Vacc+RMD timepoint. Gene expression is represented as Z-score. ChromHMM enrichment (c) and histone marks enrichment (d) based on DEGs. Down- and up-regulated genes are shown respective to Early rebounders in comparison to Late rebounders. Dot color indicates log2 Fold-change and the dot size the -log10 p-value (hypergeometric test LOLA R/Bioconductor).

**Figure S7. GSEA DMPs between Early and Late rebound.** Pathways and BTMs enriched according to DMPs between and Early and Late rebound at time points Vacc+RMD. All the pathways have a negative NES and an adjusted p-value < 0.2 (GSEA), indicating that the majority of the genes in the pathways were hypermethylated in individuals with a Late rebound. The pathways are clustered in different groups based on function. Node color is not associated with any feature.

**Figure S8. Individual DMPs between Early and Late rebound.** (a-g) Plot showing the methylation level (Beta-value) of specific CpG positions that are differentially methylated at Vacc+RMD between Early (blue) and Late (orange) rebounders. The methylation levels of each CpG site are shown at three different time points: baseline (BSL), one week after MVA.HIVconsv (Vacc) and one week after RMD administration (Vacc+RMD). * indicates a p-value < 0.05 and ** p-value < 0.01 (Mann-Whitney test). h) shows significant correlations (Spearman’s correlation) for the DMPs shown in (a-g) with different variables: CA-RNA at Vacc+RMD (ratio HIV/TBP * 1000), VL at Vacc+ RMD (ultrasensitive viral load, HIV RNA copies/ml), Days off cART (during the MAP), time on MAP with undetectable viral load (undetectable MAP) or , time on cART at BCN02 entry.

**Figure S9. Differential methylation levels in virus integration sites in early and late rebounders.** (a) shows the methylation levels (beta-values) of cg03035859_BACH2, (b) the methylation levels (beta-values) for cg02352203_RPTOR and (c-d) the methylation levels (beta-values) of RASA3 in two different positions (cg00502469 and cg1758122). In all the graphs, the methylation levels are shown for of each CpG position at the Vacc+RMD timepoint in Early (Blue) and Late rebounders (orange). P-values make reference to the limma models to identify DMPs between Early and Late rebound at Vacc+RMD. (e) shows the Rho value and the p-value for the Spearman’s correlation test between the methylation levels (beta-value) of specific CpG positions and the proviral (HIV-1 DNA copies/10^6^ CD4+ T cells) and CA-RNA (ratio HIV/TBP * 1000) levels.
